# Comparison of six statistical methods for interrupted time series studies: empirical evaluation of 190 published series

**DOI:** 10.1186/s12874-021-01306-w

**Published:** 2021-06-26

**Authors:** Simon L. Turner, Amalia Karahalios, Andrew B. Forbes, Monica Taljaard, Jeremy M. Grimshaw, Joanne E. McKenzie

**Affiliations:** 1 School of Public Health and Preventive Medicine, Monash University, Level 4, 553 St. Kilda Road, Melbourne, VIC 3004 Australia; 2 Clinical Epidemiology Program, Ottawa Hospital Research Institute, 1053 Carling Ave, Ottawa, ON Canada; 3 School of Epidemiology and Public Health, University of Ottawa, 600 Peter Morand Crescent, Ottawa, ON K1G 5Z3 Canada; 4 Department of Medicine, University of Ottawa, Roger Guindon Hall, 451 Smyth Rd, Ottawa, ON Canada

**Keywords:** Autocorrelation, Interrupted Time Series, Public Health, Segmented Regression, Statistical Methods, Empirical study

## Abstract

**Background:**

The Interrupted Time Series (ITS) is a quasi-experimental design commonly used in public health to evaluate the impact of interventions or exposures. Multiple statistical methods are available to analyse data from ITS studies, but no empirical investigation has examined how the different methods compare when applied to real-world datasets.

**Methods:**

A random sample of 200 ITS studies identified in a previous methods review were included. Time series data from each of these studies was sought. Each dataset was re-analysed using six statistical methods. Point and confidence interval estimates for level and slope changes, standard errors, *p-*values and estimates of autocorrelation were compared between methods.

**Results:**

From the 200 ITS studies, including 230 time series, 190 datasets were obtained. We found that the choice of statistical method can importantly affect the level and slope change point estimates, their standard errors, width of confidence intervals and *p-*values. Statistical significance (categorised at the 5% level) often differed across the pairwise comparisons of methods, ranging from 4 to 25% disagreement. Estimates of autocorrelation differed depending on the method used and the length of the series.

**Conclusions:**

The choice of statistical method in ITS studies can lead to substantially different conclusions about the impact of the interruption. Pre-specification of the statistical method is encouraged, and naive conclusions based on statistical significance should be avoided.

**Supplementary Information:**

The online version contains supplementary material available at 10.1186/s12874-021-01306-w.

## Background

Randomised trials are the gold standard design for investigating the impact of public health interventions, however, they cannot always be used. For example, interventions that impact an entire country, or those that have occurred historically, may preclude the ability to randomize or include control groups [1]. An alternative non-randomised design that may be considered in such circumstances is an interrupted time series (ITS) [2–4]. In an ITS design, data are collected at multiple time points both before and after an interruption (i.e. an intervention or exposure). Modelling of the data in the pre-interruption period allows estimation of the underlying secular trend, which when modelled correctly and extrapolated into the post-interruption time period, yields a counterfactual for what would have occurred in the absence of the interruption. Differences between the counterfactual and observed data at various points post interruption can be estimated (e.g. immediate and long-term effects), having accounted for the underlying secular trend.

A characteristic of data collected over time is that the data points tend to be correlated [5]. This correlation – referred to as autocorrelation or serial correlation – can be positive (whereby data points close together in time are more similar than data points further apart) or, infrequently, negative (whereby data points close together are more dissimilar than data points further apart). Autocorrelation may be observed between consecutive data points or over longer periods of time (e.g. seasonal effects). This characteristic of the data needs to be considered when designing and analysing ITS studies. If positive autocorrelation is present, larger sample sizes are required to provide power at the desired level [6] and if autocorrelation is not accounted for in the statistical analysis, standard errors may be underestimated [7].

Segmented linear regression models are often fitted to ITS data using a range of estimation methods [8–11]. Commonly ordinary least squares (OLS) is used to estimate the model parameters [10]; however, the method does not account for autocorrelation. Other statistical methods are available that attempt to account for autocorrelation in different ways (e.g. correction of standard errors, directly modelling the errors).

Turner et al. undertook a statistical simulation study examining the performance of statistical methods for analysing ITS data, where the methods were those commonly used in practice or had shown potential to perform well [12]. This simulation study provided insight into how these statistical methods performed under different scenarios, including different level and slope changes, varying magnitudes of underlying autocorrelation and series lengths. In combination with these findings, evidence from an empirical evaluation can provide a more comprehensive understanding of how the methods operate. In particular, empirical evaluations – in which methods are applied to real-world data sets and the results are compared – allow assessment of whether the choice of method matters in practice, and the degree to which they may do so.

To our knowledge, there has been no study that has empirically compared different methods for analysing ITS data when applied to a large sample of real-world data sets. We therefore undertook such an evaluation, where we aimed to compare level and slope change estimates, their standard errors, confidence intervals and *p-*values, and estimates of autocorrelation, obtained from the set of statistical methods used in the Turner et al. simulation study [12].

## Methods

### Repository of ITS studies

A sample of 200 ITS studies identified in a previous methods review were eligible for inclusion in the current study [10]. In brief, ITS studies were identified from a search of the bibliometric database PubMed between the years 2013 and 2017. Studies were stratified by year, assigned random numbers, sorted (in ascending order) by these numbers, and screened until we identified 40 studies that met the eligibility criteria. The criteria for inclusion were: 1) studies in which there were at least two segments separated by a clearly defined interruption with at least three points in each segment; 2) observations were collected on a group of individuals at each time point; and 3) the study investigated the impact of an interruption that had public health implications.

For each of the 200 studies, the first reported ITS of each outcome type (binary, continuous, count or proportion) was included, resulting in 230 ITS. Data were collected on the study characteristics and design of the ITS studies, types of outcomes, models used, statistical methods employed, effect measures reported, and the properties of included graphs. Further details of the study methods are available in the study protocol and results papers [10, 13].

### Methods to obtain time series data

Time series data from the included studies were obtained using three methods. First, we collated datasets that were reported in the published paper or its supplement (e.g. time series data reported in tables, or as text files). Second, we contacted all authors for whom we were able to obtain contact details to request datasets. We requested only aggregate level data (i.e. not individual participant data) and in the circumstance where a study included multiple series, we only sought data from the first time series reported in the paper to reduce respondent burden. We sent an initial email request on the 13^th^ December 2018 and a follow-up email on the 24^th^ January 2019. Third, we digitally extracted datasets from published graphs using the software WebPlotDigitizer [14]. This graphical data extraction tool has been found to accurately estimate the position of points on a graph [15].

If multiple datasets from the above methods were available for a particular time series, we selected the dataset generated using the following hierarchy: (i) published data, (ii) contact with authors, and (iii) digitally extracted. We checked the data provided by authors against the information reported in the publication. Where there was a discrepancy, we re-contacted the authors to query the provided data.

### Interrupted time series model

**Fig. 1:**
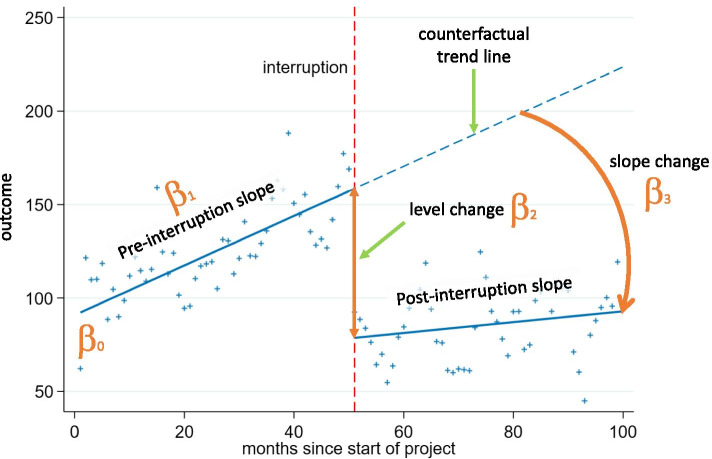
Graphical depiction of a segmented linear regression model fitted to ITS data. Secular trends (indicated by solid blue lines) for the pre and post interruption periods (indicated by the vertical dashed line) are estimated from the data (indicated by blue crosses). A counterfactual trend line (extrapolation of the pre-interruption trend line shown as a dashed blue line) is compared with the post interruption trend to estimate the immediate and longer term impact of the interruption. Model parameters are indicated as the intercept (${\beta }_{0}$); pre-interruption slope (${\beta }_{1}$); change in level at the interruption (${\beta }_{2}$), and the change in slope (${\beta }_{3}$)

We fitted segmented linear regression models to each dataset using the parameterisation of Huitema and McKean [7] (Eq. 1, Fig. 1):

1 $${Y}_{t}={\beta }_{0}+{\beta }_{1}t+{\beta }_{2}{D}_{t}+{\beta }_{3}\left[t-{T}_{I}\right]{D}_{t}+{\varepsilon }_{t}$$

where ${Y}_{t}$ represents the outcome that is measured at time point t of N time points (1 to ${n}_{1}$ measurements during the pre-interruption stage, and ${n}_{1}+1$ to ${n}_{2}$ measurements in the post-interruption stage), with the interruption occurring at time ${T}_{I}$. ${D}_{t}$ is an indicator variable that represents the post-interruption interval: coded as 0 in the pre-interruption period, and as 1 in the post-interruption period. The model parameters ($\beta$ s) represent the baseline intercept (${\beta }_{0}$); pre-interruption slope (${\beta }_{1}$); change in level at the interruption (${\beta }_{2}$), and the change in slope (${\beta }_{3}$). The model can be extended to accommodate more than one interruption with the inclusion of terms representing additional segments.

The error term ${\varepsilon }_{t}$ allows for deviation from the fitted model. In a first order (lag-1) autocorrelation model, the error at time point t (${\varepsilon }_{t}$) is influenced by only the previous data point as ${\varepsilon }_{t}=\rho {\varepsilon }_{t-1}+{w}_{t}$, where $\rho$ is the magnitude of autocorrelation (ranging from -1 to 1) and ${w}_{t}$ represents normally distributed “white noise” ${w}_{t}\sim N\left(0,{\sigma }^{2}\right)$. Longer lags can be modelled or accommodated, but here we restrict our focus to lag-1.

### Interrupted time series analysis methods

Six statistical methods were used to analyse the ITS datasets assuming first order autocorrelation (lag-1) (Table 1). The methods were chosen because they have commonly been used in practice [8–11] or because of they have been shown (through numerical simulation) to have improved confidence interval coverage relative to the methods commonly used in practice [12]. The methods were:

- ordinary least squares regression (OLS), which provides no adjustment for autocorrelation, and in the presence of positive autocorrelation will yield standard errors that are too small [16];
- OLS with Newey-West standard errors (NW), which yield OLS estimates of the model regression parameters, but with standard errors that are adjusted for autocorrelation [17];
- Prais-Winsten (PW), a generalised least squares method, which provides an extension of OLS where the assumption of independence across observations is relaxed [18, 19];
- restricted maximum likelihood (REML) (with and without the small sample Satterthwaite approximation (Satt)), which addresses bias in maximum likelihood estimators of variance components by separating the log-likelihood into two terms (one of which is only dependent on variance parameters) and using the appropriate number of degrees of freedom (d.f.) [20, 21]; and,
- autoregressive integrated moving average (ARIMA), which explicitly models the influence of previous time points by including regression coefficients from lagged values of the dependent variable and errors [22].

**Table 1:** Statistical methods, adjustments for autocorrelation and abbreviations used

Statistical method	Autocorrelation adjustment	Abbreviation
Ordinary least squares	None	OLS
	Newey-West standard error adjustment with lag-1 autocorrelation	NW
Generalised least squares	Prais-Winsten	PW
Restricted maximum likelihood	Lag-1 autocorrelation model	REML
	Lag-1 autocorrelation model with small sample Satterthwaite approximation	REML-Satt
Autoregressive integrated moving average	Lag-1 autocorrelation model (i.e. ARIMA(1,0,0))	ARIMA

### Analysis of the ITS datasets

We implemented the segmented linear regression model (Eq. 1, Sect. 2.3) by setting up datasets for each ITS study with the following variables:

- outcome variable;
- time variable t, beginning at 1 and incrementing by 1 up to time point N;
- an interruption time indicator ${D}_{t}$; coded 0 pre-interruption and 1 post-interruption; and,
- a slope change variable $\left[t-{T}_{I}\right]{D}_{t}$ *,* equal to zero at the time of the interruption (${T}_{I}$) and incrementing by 1 up to time point N.

We used information provided in the corresponding manuscript to determine the interruption time. In studies with multiple interruptions, we only included the first interruption (and adjacent periods). In studies with a transition period, we extended the model to include an additional segment for the transition period; however, when calculating the level and slope changes, we ignored this segment (further details available in Additional file 3: Appendix 1).

We analysed each dataset using the six estimation methods described in Sect. 2.4. For REML with the Satterthwaite approximation, when the computed degrees of freedom were less than two, we substituted these with the value two to avoid overly conservative confidence limits and hypothesis tests. We only included analyses for which the estimate of autocorrelation was strictly between -1 and + 1. The datasets were analysed in Stata 15 [23] (see Additional file 1 for analysis code).

### Comparison of results from the different ITS analysis methods

The results of interest were point estimates of the immediate level change (β_2_) and slope change (β_3_), their associated standard errors, confidence intervals and *p-*values, and the estimated lag-1 autocorrelation. Across the ITS studies, different outcomes were measured, necessitating the need to standardise the estimates of slope and level change for comparison across the datasets. This was achieved for each dataset by dividing parameter estimates by the root mean square error (RMSE) estimated from a segmented linear regression model using OLS. We also standardised the direction of effect. This was achieved for each pairwise comparison of methods by multiplying both estimates by -1 if the first method’s estimate was less than zero. We also repeated these analyses standardising to the direction of the second method’s estimate.

#### Estimates of level and slope changes, and their standard errors

We compared the level and slope change point estimates with their standard errors using visual displays and tabulation. Specifically, we used Bland Altman scatter plots [24] to assess pairwise agreement in the results (standardised estimates of level change, slope change, and their standard errors) between the different statistical methods. For each pairwise comparison, the difference in the two estimates was plotted against the average of the two estimates (e.g. ‘difference in estimates of level change from OLS and PW’ versus ‘average of estimates of level change from OLS and PW’). In the case of the standard errors, we first log-transformed these to remove the relationship between the variability of the differences and the magnitude of the standard errors [24]. The mean difference and limits of agreement (average difference $\pm$ 1.96 $\times$ standard deviation of the differences) were calculated and overlaid on the plots. These pairwise comparisons were displayed in a matrix of plots to show comparisons of each method with all others. Plots in the top triangle of the matrix illustrate agreement between the effect estimates (either level change or slope change), and plots in the bottom triangle illustrate the agreement between the standard errors.

We also investigated whether series length impacted the difference in level and slope change estimates between each pair of methods. A matrix of scatterplots of the differences versus the (log) length of series (overlaid with a local regression (LOESS) smoothed curve) for each pairwise method comparison was used to visually examine this relationship.

#### Confidence Intervals

We visually compared the width of the confidence intervals from the different statistical methods. For each dataset and pairwise comparison, a ratio of the confidence interval widths from the two methods was calculated and then scaled so that the comparison method confidence interval spanned -0.5 to 0.5.

#### *p-*values

We compared the *p-*values of the effect estimates between the methods by categorising the *p-*values based on commonly used levels of statistical significance. First, we categorised the *p-*values at the 5% level of statistical significance (i.e. < 5%, ≥ 5%), and second, we categorised *p-*values using a finer gradation (i.e. *p-*value < 1%, 1% ≤ *p-*value < 5%, 5% ≤ *p-*value < 10%, *p-*value ≥ 10%). For each pairwise comparison between methods, we calculated the percentage of datasets where there was agreement in the categories of statistical significance (i.e. the percentage of datasets where the *p-*value for the effect estimate was < 0.05 for both methods *or* the *p-*value was ≥ 0.05 for both methods). Further, we calculated kappa statistics to assess agreement beyond chance. We use the following adjectives when describing the results: 0.41–0.6 moderate agreement, 0.61–0.8 substantial agreement, 0.81–1.0 almost perfect agreement [25].

### Autocorrelation coefficient estimates

We calculated and tabulated medians and interquartile ranges for estimates of lag-1 autocorrelation for the three methods that yield these estimates (ARIMA, PW, REML). The summary statistics are reported for all series as well as being restricted to series with ≥ 24 points and series with ≥ 100 points, in order to assess whether series length impacted the magnitude of the estimates. A scatterplot of autocorrelation versus (log) length of series (overlaid with a LOESS curve) was used to visually examine this relationship. A further scatter plot was generated that depicted the REML estimates of autocorrelation along with their confidence intervals.

## Results

### Time series dataset acquisition

**Fig. 2:**
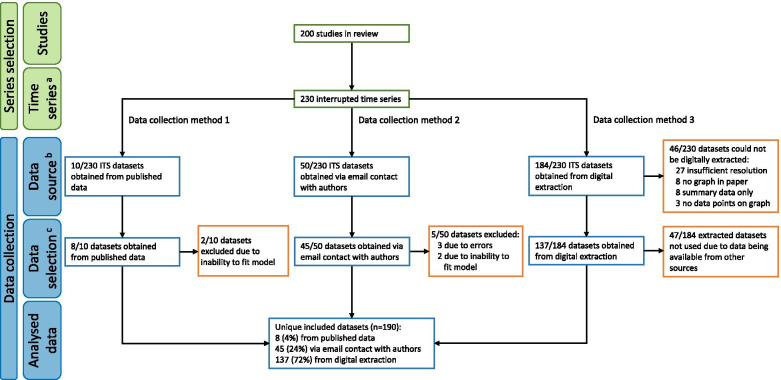
Flowchart of selected datasets. Green boxes denote the number of included studies and time series, blue boxes denote the numbers corresponding to dataset collection, and orange boxes denote the numbers corresponding to dataset exclusion.^a^ Some studies included multiple interrupted time series, hence the number of time series is greater than the number of studies. ^b^ As multiple methods were potentially available for obtaining an interrupted time series dataset (e.g. some datasets were obtained via both email contact and digital extraction), the numerators across the data sources do not sum to 230. ^c^ For each interrupted time series, only one data source was selected for analysis, yielding a total of 190 unique time series datasets. The hierarchy for the data source selection was (i) published data, (ii) contact with authors, and (iii) digital extraction

Of the 230 ITS identified in the review [10] we obtained 10/230 (4%) datasets directly from the publication (e.g. time series data reported in tables), 50/230 (22%) through email contact with the authors, and 184/230 (80%) through digital data extraction. For some series (n = 47), multiple datasets from the different sources were available (Fig. 2). Using our hierarchy for selecting the source of the dataset when multiple series were available resulted in 190 unique datasets, with 8/190 (4%) sourced directly from the publication, 45/190 (24%) through email contact with authors, and 137/190 (72%) from digital data extraction. We were unable to obtain 40 of the 230 ITS included in the review because the data were not reported in the paper, could not be obtained from authors, or could not be digitally extracted. Five of the datasets obtained from the authors could not be used: three due to errors in the data; two because the data were too complex to fit a simple segmented linear regression model. Forty-six of the datasets could not be digitally extracted, 27 studies included graphs with insufficient resolution to digitally extract data; 8 studies had no graph; 8 studies had summary data only (e.g. a summary graph showing a small number of annual figures was provided when monthly data was used in the analysis); and 3 studies had graphs but did not plot data points.

### Characteristics of the included ITS

The characteristics of the ITS studies with available datasets for re-analysis are compared to all 200 ITS studies in Table 2. No major differences were found. The types of study interventions were similar, as were the types of time intervals. The number of time points per series were lower in the studies with available datasets than in all ITS studies (median 41, IQR [25, 71] versus 48, IQR (30, 100)). The length of the segments used to calculate the estimates for the first interruption were slightly shorter in the series with available data than in all series (16, IQR (10, 28) versus 18 IQR (10, 34)).

**Table 2:** Characteristics of interrupted time series studies and series

Study level characteristics	All ITS studies (*n* = 200)		ITS studies with available data (*n* = 166)	
	*n*	%	*n*	%
Type of interruption				
Exposure^a^	12	6	10	6
Intervention	188	94	156	94
Intervention type				
Policy change	104	52	82	49
Practice change	40	20	36	22
Communication	29	15	24	14
Organisation of care	13	7	12	7
Clinical intervention	2	1	2	1
Time interval type				
Daily	3	2	2	1
Weekly	9	5	6	4
Two weekly	1	1	1	1
Monthly	120	60	96	58
Quarterly	31	16	28	17
Six monthly	3	2	3	2
Annually	20	10	17	10
Other	12	6	12	7
Can't determine	1	1	1	1
Series level characteristics	ITS (*n* = 230)		ITS with available data (*n* = 190)	
	median	IQR	median	IQR
Number of time points per series	48	(30, 100)	41	(25, 71)
Number of time points in the segments used to calculate estimates for the first interruption	18	(10, 34)	16	(10, 28)

*Abbreviations*: *ITS* Interrupted time series, *IQR* Inter-quartile range

^a^Our definition of an exposure is limited to exposures or events that are not under investigator control (e.g. earthquakes, financial crises, tsunamis, environmental chemicals). We use the term ‘investigator’ loosely to include researchers, clinicians and policy makers

### Comparison of results from the different ITS analysis methods

#### Estimates of level and slope changes, and their standard errors

The median values of the absolute value of the standardised effect estimates for level change ranged from 1.22 to 1.49 across the statistical methods (Table 3). For slope change, the median value of the absolute value of the standardised effect estimates was 0.13 for all statistical methods (Table 3). Pairwise comparisons were limited to a minimum of 171 datasets because at least one statistical method failed to converge, failed to yield standard errors or estimated the magnitude of autocorrelation to be outside the range -1 to + 1 in 19 of the datasets (Table 4).

**Table 3:** Effect estimate summaries

	**N**	**Absolute value of effect estimate**	
		**Level change** **Median (IQR)**	**Slope change** **Median (IQR)**
ARIMA	189	1.40 (0.63,2.90)	0.13 (0.05,0.26)
OLS (NW)^a^	190	1.49 (0.60,3.03)	0.13 (0.06,0.27)
PW	189	1.33 (0.57,2.81)	0.13 (0.05,0.26)
REML (REML-Satt)^a^	181	1.22 (0.47,2.56)	0.13 (0.05,0.25)

Abbreviations: *IQR* Interquartile range, *ARIMA* Autoregressive integrated moving average, *OLS* Ordinary least squares, *PW* Prais-Winsten, *REML* Restricted maximum likelihood

^a^ The NW and OLS methods use the same estimator for level and slope change, as do REML and REML-Satt

**Table 4:** Number of available comparisons for the statistical methods investigated (*n* = 190)

Number of comparisons	ARIMA	OLS	NW	PW	REML	REML-Satt
ARIMA	189	189	188	185	175	175
OLS		190	189	186	175	175
NW			189	186	174	174
PW				186	171	171
REML					175	175
REML-Satt						175

*Abbreviations*: *ARIMA* Autoregressive integrated moving average, *OLS* Ordinary least squares, *NW* OLS with Newey-West standard error adjustments, *PW* Prais-Winsten, *REML* Restricted maximum likelihood, *REML-Satt* Restricted maximum likelihood with Satterthwaite small sample adjustment

**Fig. 3:**
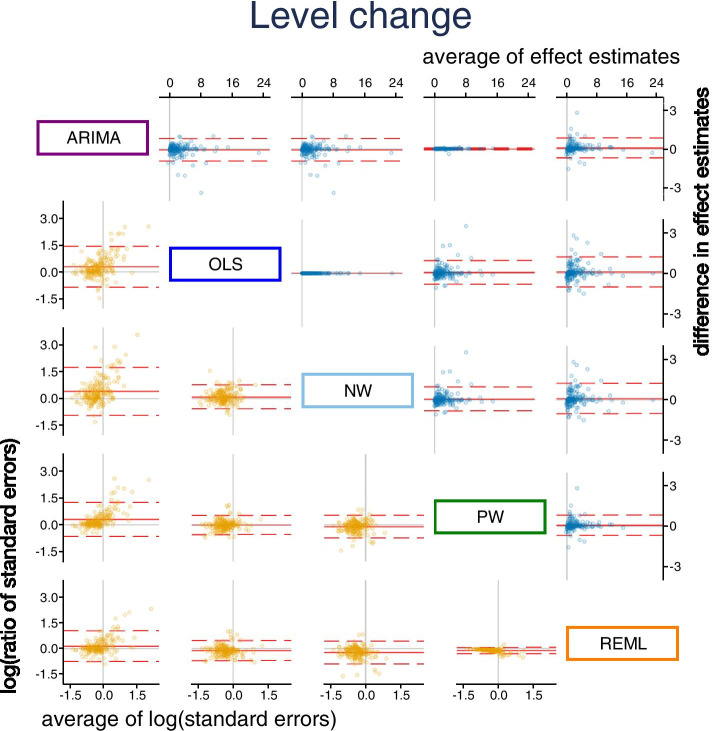
Bland Altman plot of standardised level change. Plots in the top triangle (blue points) show the difference in point estimates (row method – column method) on the vertical axis and average of the parameter estimates on the horizontal axis. Plots in the bottom triangle (orange points) show differences in standard errors on the vertical axis (= log(ratio of standard errors)) (column method – row method) and the average of the log of the standard errors on the horizontal axis. Red horizontal lines depict the average, red dashed lines depict the 95% limits of agreement (calculated as the average ± 1.96*standard deviation of the differences). Grey lines indicate zero. Abbreviations: ARIMA, autoregressive integrated moving average; OLS, ordinary least squares; NW OLS with Newey-West standard error adjustments; PW, Prais-Winsten; REML, restricted maximum likelihood. Note that REML with the Satterthwaite approximation is not presented because it only makes an adjustment to the confidence intervals, and not the standard errors

**Fig. 4:**
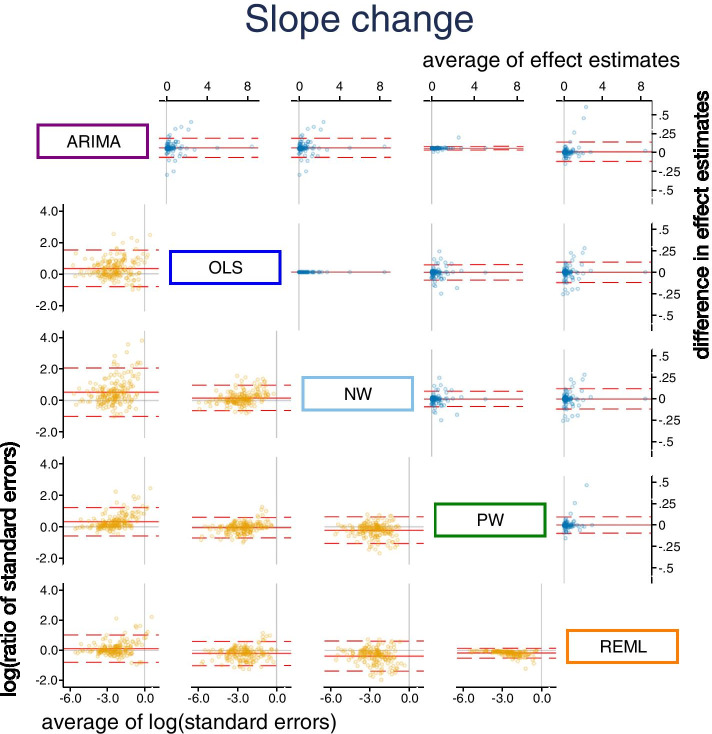
Bland Altman plot of standardised slope change. Plots in the top triangle (blue points) show the difference in point estimates (row method – column method) on the vertical axis and average of the parameter estimates on the horizontal axis. Plots in the bottom triangle (orange points) show differences in standard errors on the vertical axis (= log(ratio of standard errors)) (column method – row method) and the average of the log of the standard errors on the horizontal axis. Red horizontal lines depict the average, red dashed lines depict the 95% limits of agreement (calculated as the average ± 1.96*standard deviation of the differences). Grey lines indicate zero. Abbreviations: ARIMA, autoregressive integrated moving average; OLS, ordinary least squares; NW OLS with Newey-West standard error adjustments; PW, Prais-Winsten; REML, restricted maximum likelihood. Note that REML with the Satterthwaite approximation is not presented because it only makes an adjustment to the confidence intervals, and not the standard errors

Pairwise comparisons of level change, slope change, and their standard errors for each of the five methods were made (Figs. 3 and 4). REML with the Satterthwaite approximation was excluded from these comparisons because it only adjusts the width of the confidence intervals, and not the standard errors. There were small systematic differences in estimates of level change in the pairwise comparisons between the methods, REML had slightly smaller and OLS slightly larger effect estimates than the other methods (Fig. 3, top triangle, and Table 5). The largest limits of agreement between all methods (REML vs OLS) were ± 1.11. Expectedly, there was no difference in the standardised level change estimates between OLS and NW (since they use the same estimator for ${\beta }_{2}$) and a very small difference between PW and ARIMA (since their point estimation methods are almost equivalent). There were no systematic differences in slope change estimates between the methods (Fig. 4, top triangle and Table 6). Limits of agreement for slope change were generally similar across the pairwise comparisons of methods (but again with the exceptions of the comparison between OLS and NW, and PW and ARIMA).

There were systematic differences in the estimates of standard error of level change across some pairwise comparisons of methods (Fig. 3, bottom triangle, and Table 5). Notably, the ARIMA standard errors were systematically larger compared with all other methods; however, this difference was smaller when compared with REML (geometric mean ratio standard errors for level change of 1.15). Aside from the pairwise comparison between PW and REML, the limits of agreement between the methods showed that the methods could yield large differences in the standard errors, particularly so for ARIMA compared with the other methods. For example, the limits of agreement for ARIMA compared with NW showed that the differences in standard errors could be large, ranging from 61% smaller to 460% larger. Similar patterns were observed for slope change (Fig. 4 bottom triangle, and Table 6).

**Table 5:**
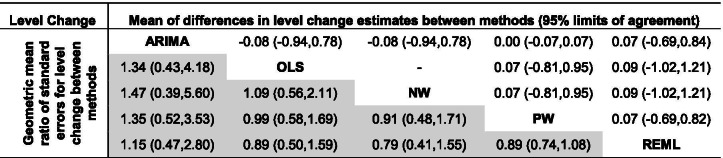
Mean of differences in level change estimates between methods (row method-column method) (top triangle) and geometric mean ratio of standard errors for level change between methods (column method/row method) (shaded bottom triangle) with 95% limits of agreement. The NW and OLS methods use the same estimator for level and slope change, as do REML and REML-Satt (not shown), which also use the same estimator for standard errors

**Table 6:**
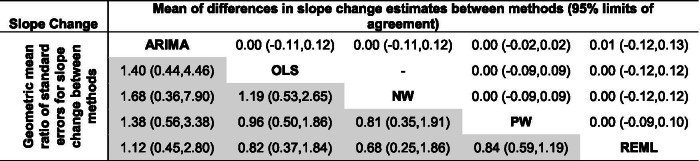
Mean of differences in slope change estimates between methods (row method—column method) (top triangle) and geometric mean ratio of standard errors for slope change between methods (column method/row method) (shaded bottom triangle) with 95% limits of agreement. The NW and OLS methods use the same estimator for level and slope change, as do REML and REML-Satt (not shown), which also use the same estimator for standard errors

Our visual examination of the impact of series length on the differences in level change estimates between pairs of methods showed that series length was not associated with the differences, with the exception of comparisons with the REML method. For these comparisons, the variability of the differences decreased for longer series (Additional file 3: Appendix 2). The variability in differences in slope change estimates for all pairwise comparisons between methods (except between ARIMA and PW), tended to decrease with increasing series length.

When we repeated the analysis standardising the direction of effect to the second method’s estimate, we found the results did not importantly change (Additional file 3: Appendix 3).

**Table 7:**
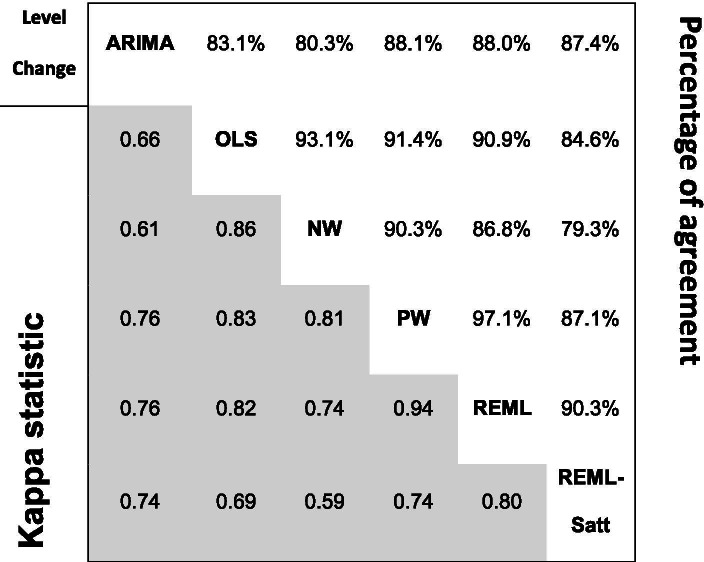
Pairwise agreement in statistical significance of estimates of level change between statistical methods. *P*-values associated with estimates of level change were categorised at the 5% level of statistical significance (i.e. <5%, ≥5%). Cells in the upper triangle contain the percentage of series for which the *p*-value for level change was < 0.05 for both methods or the *p*-value was ≥0.05 for both methods. Denominators are reported in Table 4. Cells in the lower triangle (shaded) contain kappa statistics. Abbreviations: ARIMA, autoregressive integrated moving average; OLS, ordinary least squares; NW, OLS with Newey-West standard error adjustments; PW, Prais-Winsten; REML, restricted maximum likelihood; REML-Satt, restricted maximum likelihood with Satterthwaite small sample adjustment

#### Confidence Intervals

**Fig. 5:**
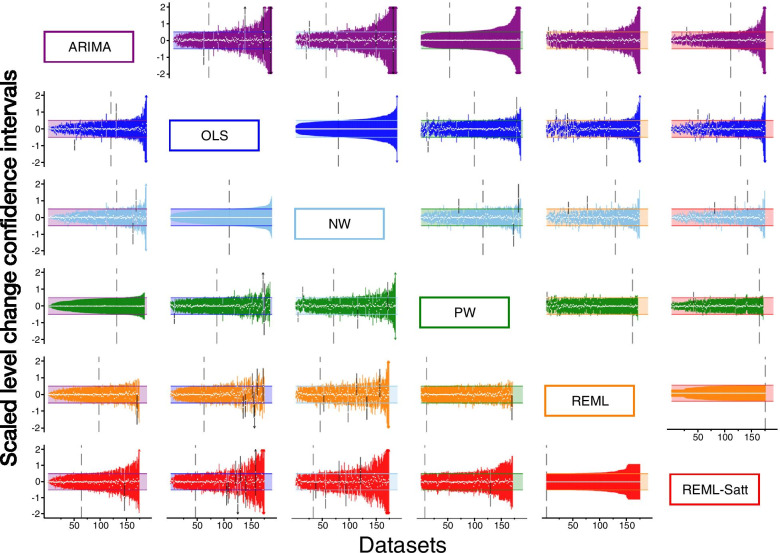
Pairwise confidence interval comparisons for level change. Each plot displays up to 190 confidence intervals (CIs) (depicted as vertical lines), with each scaled so that the confidence interval from the reference method spans -0.5 to 0.5 (shaded area). The reference method is the column method (e.g. the plot in the second row, first column shows OLS CIs (blue) compared to ARIMA (purple)). Vertical lines falling entirely within the shaded area have smaller confidence intervals than the comparison (left of the vertical dashed line), while lines extending beyond the shaded area have larger confidence intervals than the comparison (right of the vertical dashed line). White dots indicate the point estimate. Black vertical lines indicate scenarios in which the point estimate from one method does not lie within the confidence interval of the other. Abbreviations: ARIMA, autoregressive integrated moving average, purple; OLS, ordinary least squares, blue; NW OLS with Newey-West standard error adjustments, light blue; PW, Prais-Winsten, light green; REML, restricted maximum likelihood, orange; REML-Satt, restricted maximum likelihood with Satterthwaite small sample adjustment, red

**Fig. 6:**
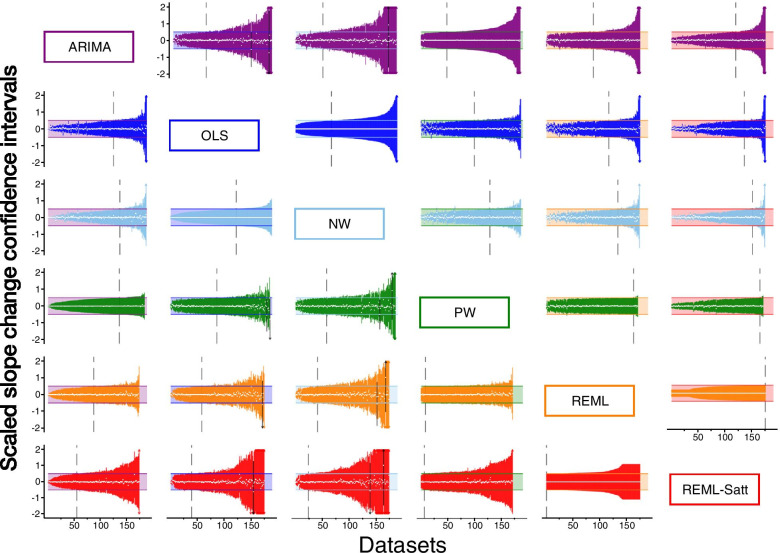
Pairwise confidence interval comparisons for slope change. Each plot displays up to 190 confidence intervals (CIs) (depicted as vertical lines), with each scaled so that the confidence interval from the reference method spans -0.5 to 0.5 (shaded area). The reference method is the column method (e.g. the plot in the second row, first column shows OLS CIs (blue) compared to ARIMA (purple)). Vertical lines falling entirely within the shaded area have smaller confidence intervals than the comparison (left of the vertical dashed line), while lines extending beyond the shaded area have larger confidence intervals than the comparison (right of the vertical dashed line). White dots indicate the point estimate. Black vertical lines indicate scenarios in which the point estimate from one method does not lie within the confidence interval of the other. Abbreviations: ARIMA, autoregressive integrated moving average, purple; OLS, ordinary least squares, blue; NW OLS with Newey-West standard error adjustments, light blue; PW, Prais-Winsten, light green; REML, restricted maximum likelihood, orange; REML-Satt, restricted maximum likelihood with Satterthwaite small sample adjustment, red

Pairwise comparisons of the confidence interval width for the estimated level change between the methods reflected the patterns observed when comparing the standard errors (Fig. 5). ARIMA generally yielded wider confidence intervals with 64%, 70% and 71% of the ARIMA confidence intervals being wider than OLS, NW and PW respectively. ARIMA confidence intervals widths were similar to REML. REML with the Satterthwaite confidence interval adjustment yielded the widest confidence intervals of all methods; only 37% of ARIMA confidence intervals were wider than REML with Satt. This pattern was also seen when comparing the confidence interval widths for the estimated slope change between the methods (Fig. 6).

#### *p-*values

**Fig. 7:**
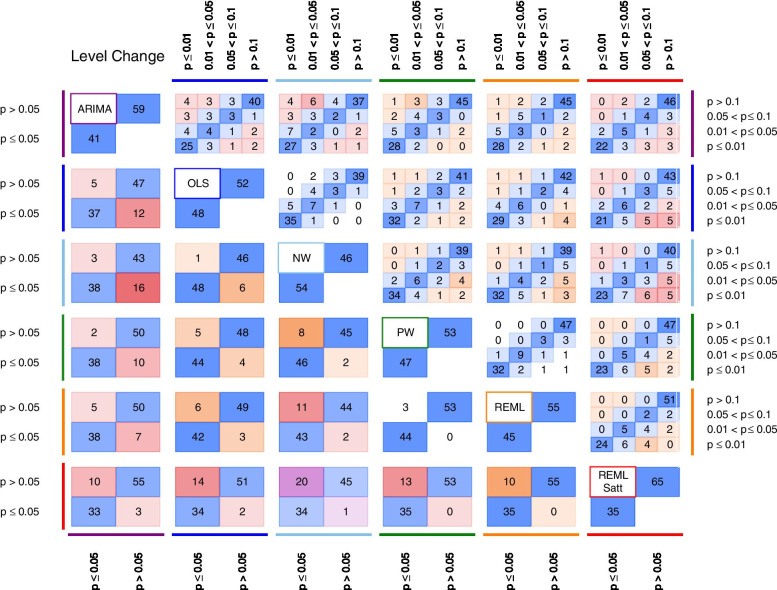
Pairwise agreement in statistical significance of estimates of *p-*value comparisons for level change. In the top triangle, boxes are divided into 16 cells with *p-*values categorised using a fine gradation of statistical significance, namely, *p-*value ≤ 0.01, 0.01 < *p-*value ≤ 0.05, 0.05 < *p-*value ≤ 0.1, *p-*value > 0.1. In the bottom triangle, boxes are divided into four cells with *p-*values categorised at the 5% level of statistical significance (i.e. ≤ 5%, > 5%). Each cell within a box contains the percentage of datasets falling within the row and column defined statistical significance levels. The colour bands surrounding the left/right and top/bottom side of the plot indicate the two methods being compared. Concordant results are shown in blue. Discordant results are shown as either white (0–5% discordance), orange (5–10% discordance), red (10–20% discordance) or purple (over 20% discordance). For example, within the box comparing ARIMA and OLS in the bottom triangle, in 12% of the datasets the ARIMA method yields a *p-*value > 0.05 while the OLS method yields a *p-*value ≤ 0.05 (bottom right cell). Numbers may not add to 100 due to rounding. Abbreviations: ARIMA, autoregressive integrated moving average; OLS, ordinary least squares; NW OLS with Newey-West standard error adjustments; PW, Prais-Winsten; REML, restricted maximum likelihood; Satt, Satterthwaite adjustment

The percentage agreement in statistical significance (dichotomised at the 5% significance level) for level change in the pairwise comparisons between methods ranged from 79.3% (NW versus REML-Satt) to 97.1% (PW versus REML) (Table 7). Corresponding kappa statistics ranged from 0.59 (moderate agreement) for NW versus REML-Satt to 0.94 (almost perfect agreement) for PW versus REML. Discordance in statistical significance in comparisons with REML-Satt and ARIMA arose because these methods yielded larger *p-*values (Fig. 7). For example, in the comparison of NW with REML-Satt, 20% of NW analyses yielded a *p-*value ≤ 0.05 when the REML-Satt *p-*value was > 0.05, while only 1% of NW analysis yielded a *p-*value > 0.05 when the REML-Satt *p-*value was ≤ 0.05.

**Fig. 8:**
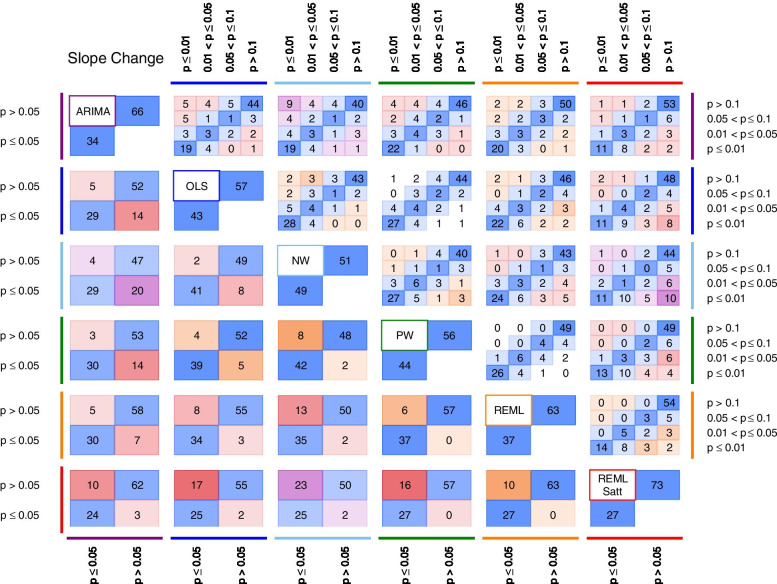
Pairwise agreement in statistical significance of estimates of *p-*value comparisons for slope change. In the top triangle, boxes are divided into 16 cells with *p-*values categorised using a fine gradation of statistical significance, namely, *p-*value ≤ 0.01, 0.01 < *p-*value ≤ 0.05, 0.05 < *p-*value ≤ 0.1, *p-*value > 0.1. In the bottom triangle, boxes are divided into four cells with *p-*values categorised at the 5% level of statistical significance (i.e. ≤ 5%, > 5%). Each cell within a box contains the percentage of datasets falling within the row and column defined statistical significance levels. The colour bands surrounding the left/right and top/bottom side of the plot indicate the two methods being compared. Concordant results are shown in blue. Discordant results are shown as either white (0–5% discordance), orange (5–10% discordance), red (10–20% discordance) or purple (over 20% discordance). For example, within the box comparing ARIMA and OLS in the bottom triangle, in 14% of the datasets the ARIMA method yields a *p-*value > 0.05 while the OLS method yields a *p-*value ≤ 0.05 (bottom right cell). Numbers may not add to 100 due to rounding. Abbreviations: ARIMA, autoregressive integrated moving average; OLS, ordinary least squares; NW OLS with Newey-West standard error adjustments; PW, Prais-Winsten; REML, restricted maximum likelihood; Satt, Satterthwaite adjustment

In general, the agreement was less for slope change compared with level change (Table 8). The percentage agreement in statistical significance (at the 5% significance level) for slope change in the pairwise comparisons between methods ranged from 75.3% (NW versus REML-Satt) to 93.6% (PW versus REML). Corresponding kappa statistics ranged from 0.50 (moderate agreement) for NW versus REML-Satt to 0.87 (almost perfect agreement) for PW versus REML. The direction of disagreement was similar to that of level change with ARIMA and REML-Satt methods yielding larger *p-*values more often than the other methods (Fig. 8).

Our examination of agreement using a finer gradation of statistical significance categories showed that when there was discordance between methods, this generally occurred in an adjacent category (e.g. one method with a *p-*value ≤ 0.01 and the comparison method with 0.01 ≤ *p-*value < 0.05). However, there were some examples where there was discordance in non-adjacent categories. For level change these comparisons were ARIMA versus NW, NW versus REML-Satt, and OLS versus REML and REML-Satt (Fig. 7), while for slope change these comparisons were the same, but also with the addition of PW versus REML-Satt (Fig. 8). The *p-*values yielded from ARIMA and REML-Satt were generally larger than the other methods, and by contrast, the *p-*values for NW, and to a lesser extent OLS, tended to be smaller (Additional file 3: Appendix 4).

**Table 8:**
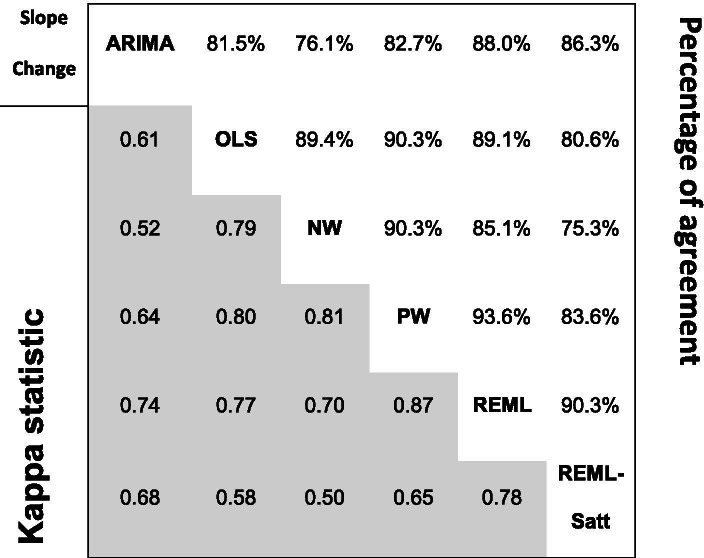
Pairwise agreement in statistical significance of estimates of slope change between statistical methods. *P*-values associated with estimates of level change were categorised at the 5% level of statistical significance (i.e. ≤ 5%, > 5%). Cells in the upper triangle contain the percentage of series for which the *p*-value for level change was ≤ 0.05 for both methods or the *p*-value was > 0.05 for both methods. Denominators are reported in Table 4. Cells in the lower triangle (shaded) contain kappa statistics. Abbreviations: ARIMA, autoregressive integrated moving average; OLS, ordinary least squares; NW, OLS with Newey-West standard error adjustments; PW, Prais-Winsten; REML, restricted maximum likelihood; REML-Satt, restricted maximum likelihood with Satterthwaite small sample adjustment

### Autocorrelation coefficient estimates

**Fig. 9:**
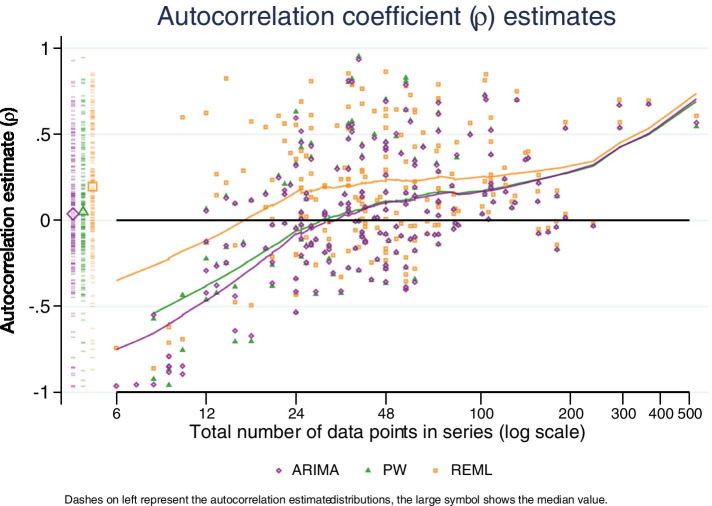
Autocorrelation coefficient estimates. Scatterplot showing the autocorrelation estimate on the vertical axis and length of data series on the (log scale) horizontal axis. LOESS lines are overlaid to show trends in autocorrelation coefficient with data series length. Dashed lines on the left show the distribution of the estimates with overlaid symbols showing the median value. Abbreviations: ARIMA, autoregressive integrated moving average; PW, Prais-Winsten; REML, restricted maximum likelihood

Three of the statistical methods (ARIMA, PW, REML) yielded estimates of autocorrelation (Table 9, Fig. 9). The REML method estimated consistently larger magnitudes of autocorrelation than the other methods (median and inter-quartile range (IQR) of 0.2 (-0.01, 0.54) compared with 0.04 (-0.15, 0.30) for ARIMA and 0.05 (-0.14, 0.33) for PW). When restricting the examination of autocorrelation to datasets where all three methods could be compared (n = 171 datasets), the summary statistics were essentially unchanged.

**Table 9:** Autocorrelation coefficient estimates (REML estimates of -1 and 1 are excluded, PW estimates < -1 are excluded)

**Statistical method**	**Autocorrelation coefficient (ρ) estimate**					
	**All available datasets**		**Series with ≥ 24 points**		**Series with ≥ 100 points**	
	***N***	**median (IQR)**	***N***	**median (IQR)**	***N***	**median (IQR)**
ARIMA	189	0.04 (-0.15,0.30)	154	0.07 (-0.10,0.36)	31	0.19 (0.04,0.54)
PW	186	0.05 (-0.14,0.33)	155	0.07 (-0.10,0.38)	31	0.19 (0.04,0.54)
REML	175	0.20 (-0.01,0.54)	147	0.20 (-0.01,0.53)	31	0.23 (0.08,0.57)
**Restricted to datasets where all methods can be compared**						
ARIMA	171	0.05 (-0.14,0.30)	147	0.06 (-0.11,0.35)	31	0.19 (0.04,0.54)
PW	171	0.05 (-0.14,0.31)	147	0.07 (-0.11,0.35)	31	0.19 (0.04,0.54)
REML	171	0.20 (-0.01,0.54)	147	0.20 (-0.01,0.53)	31	0.23 (0.08,0.57)

The difference between REML and the other methods was more pronounced for shorter series (Table 9, Fig. 9). All methods tended to yield negative values for short data series (fewer than approximately 12 data points). In longer data series (≥ 100 data points) all methods yielded similar estimates.

**Fig. 10:**
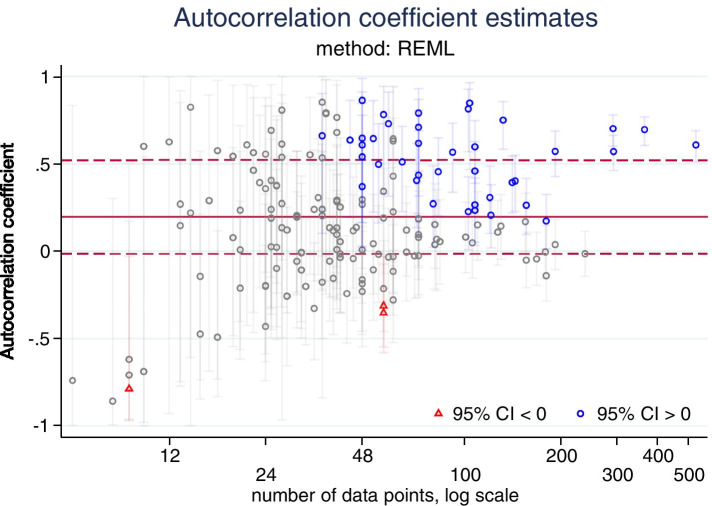
Autocorrelation coefficient estimates using the restricted maximum likelihood (REML) method. Data from 172 datasets. Red horizontal lines show the median and IQR of 0.2 (-0.02, 0.52). Blue circular markers indicated 95% confidence intervals that lie entirely above zero, red triangular markers indicate 95% confidence interval that lie entirely below zero

Confidence intervals for the REML estimates of autocorrelation show that for most studies with fewer than 48 data points the confidence limits extend below and above zero (Fig. 10). For longer series, as expected, the confidence intervals are narrow, with many excluding no and negative autocorrelation estimates.

## Discussion

### Summary and discussion of key findings

We re-analysed 190 ITS using six statistical methods and compared estimates of immediate level change, slope change, their associated standard errors, confidence intervals and *p-*values, and the estimated lag-1 autocorrelation. We found important inconsistency in these estimates across the methods, such that the interpretation of the findings in some series may differ depending on the chosen method.

On average, there were small systematic differences in estimates of level change across the statistical methods, with OLS yielding slightly larger estimates, and REML slightly smaller estimates compared with the other methods. For slope change, all methods yielded, on average, similar estimates. For some pairwise comparisons, the limits of agreement indicated large differences could arise. This was particularly notable in the comparisons between REML and the other methods. There were systematic differences in the standard errors between most methods, and the limits of agreement also indicated large differences could arise. ARIMA yielded systematically larger standard errors compared with all other methods, although the difference with REML was not as large. Of note, the PW yielded, on average, similar standard errors as OLS. This was perhaps surprising given PW provides adjustment for autocorrelation (which OLS does not), and in a numerical simulation study investigating the performance of these methods, PW was shown to perform better than OLS for data series approximately longer than 24 points [12]. The results in our empirical investigation therefore likely reflect the influence of shorter data series.

The differences in point estimates and standard errors led to differences in the confidence interval widths, *p-*values, and statistical significance. Reflecting the pattern observed with standard errors, the ARIMA confidence intervals were wider compared with the other methods. However, REML with the Satterthwaite adjustment, which adjusts the t-distribution degrees of freedom used in the calculation of the confidence interval to account for uncertainty in estimation of the standard error, yielded the widest confidence intervals.

Our results show that naively basing conclusions on statistical significance could lead to a qualitatively different interpretation. There was important discordance in statistical significance (at the 5% level) across many of the pairwise method comparisons. As expected, the discordance was greatest between the methods that yielded larger standard errors or adjusted for uncertainty in estimation of the standard error (i.e. ARIMA, and REML with SW, respectively) and the other methods.

For long series (≥ 100 data points), all methods yielded similar estimates of autocorrelation. The methods yielded different estimates with short to medium length series (i.e. < 100 data points), with the ARIMA and OLS autocorrelation estimates being substantially smaller than REML. For any ITS, we can conceive that the data collected and analysed in the study is a subset of a much longer series. If we make the assumption that there is a stable true underlying autocorrelation, then autocorrelation estimated from different series lengths, should be similar. We generally found this to be the case for REML; however, for ARIMA and PW, estimates of autocorrelation were notably smaller in short series compared with long series. This suggests that ARIMA and PW may be problematic for short series. The stability of REML estimates over the different series lengths is suggestive of it being the preferable estimator, which has been shown in numerical simulation studies to be the case [12, 26].

The magnitude of autocorrelation estimates from these ITS public health datasets, with a median of 0.23 (IQR 0.08 to 0.57, restricted to series with ≥ 100 data points, n = 31 REML method), indicate that autocorrelation should not be ignored in the design or analysis of ITS studies. Despite this, in nearly 50% (113/230) of the series included in the review, autocorrelation was not considered, or the method to adjust for autocorrelation could not be determined [10]. Furthermore, only 1.5% (3/200) of studies provided evidence of a sample size calculation, and only two of these considered autocorrelation. Similar findings have also been observed in other systematic reviews. Jandoc et al. [8] found that only 146/220 (66.4%) ITS studies reported testing for autocorrelation, Hudson et al. [11] found that 63/115 (55%) considered autocorrelation, Ewusie et al. [9] found that only 812/1365 (59.5%) checked for autocorrelation and Hategeka et al. [27] similarly found that 66/120 (55%) checked or adjusted for autocorrelation.

### Strengths and limitations

There are several strengths to our study. First, the repository of ITS studies was randomly sampled from PubMed, thus the findings are likely to be generalisable to ITS studies indexed in this database. Second, we used a variety of methods to obtain the time series data (primarily digital data extraction [14]) to optimise the number of datasets retrieved, which resulted in a large percentage of datasets being retrieved (190/230; 83%). Finally, we investigated a range of statistical methods, including those commonly used in practice [8–11], and compared their results using metrics of interest to researchers (point estimates, standard errors, confidence intervals, *p-*values, statistical significance) to provide a comprehensive picture of how the methods compared.

One limitation of this study is that our findings may not be generalisable to ITS studies outside of public health. For example, this would be the case if influencing characteristics (e.g. series length) of ITS studies in public health differ to other disciplines. Another limitation is that although the methods we included are those that are commonly used in practice [10], other methods are available (for example, forecast [28] or Bayesian [29] methods). We purposely excluded the Cochrane-Orcutt method (which is used in practice [10]), because the PW method is essentially the Cochrane-Orcutt method, except that the PW method retains the first observation, and so is advantageous for short time series [19]. A further limitation of our study is that we fitted a segmented linear regression model, assuming a continuous data type with lag-1 autocorrelation, to all datasets. This model may have differed to that used in the original publication, and furthermore, may not have been the best fitting model. However, our re-analysis was not intended to specifically address the research question(s) of the original publications, but as a means of comparing different statistical methods.

### Implications for practice

Our research has shown that in this set of ITS studies, the choice of statistical method can importantly affect the findings. This could lead to ‘bias in the selection of the reported result’ [30], where the reported result is chosen based on its magnitude, direction of effect, or statistical significance. Publication of protocols with detailed statistical analysis plans provide a mechanism for study authors to engender trust in the reported results (i.e. when there is consistency between the planned and used analysis methods). Protocols also allow readers to assess whether there were any changes to the analysis, and if so, what the legitimacy of those changes were.

Protocols should include specification of the primary analysis method, and may include a set of sensitivity analyses that allow examination of the robustness of the findings (e.g. level and slope change estimates and their confidence intervals) to the chosen analysis method. The primary analysis method needs to be carefully chosen considering characteristics of the ITS. For example, Turner et al. [12] found through a numerical simulation study that the length of the series is an important factor for deciding on the statistical method. Sensitivity analyses may be particularly important for short series, where estimates from the methods are likely to be most different.

While protocols and statistical analysis plans are now common for randomised trials [31], in our review of ITS studies, none of the 200 studies reported having a published protocol. Protocols can be published in a peer-reviewed journal, published on a pre-print server (e.g. medRxiv), or registered in an online registry (e.g. open science framework).

Finally, we recommend that time series data, including dates of the interruptions and any transition periods be made available alongside the publication. At a minimum, any plots of ITS data should follow graphing recommendations [32] to facilitate data extraction using digitising software [14].

### Implications for future research

Future research examining factors that may modify the magnitude of autocorrelation (e.g. type of outcome) would be useful. Knowledge of these factors would facilitate informed predictions about the likely magnitude of autocorrelation for an individual ITS study with particular characteristics, which could be used to more accurately determine the required sample size. Similar research has been undertaken investigating factors that modify intra-cluster correlations (ICCs) in cluster randomised trials, which has led to generalizable ‘rules-of-thumb’ on the selection of ICCs for sample size calculations in cluster trials [33].

## Conclusion

ITS studies are commonly used in public health research to assess the impact of an intervention or exposure. A range of statistical methods are available to analyse ITS, and our study has shown that the choice of method can importantly affect the level and slope change estimates, their standard errors, width of confidence intervals and *p-*values. These differences may lead to qualitatively different conclusions being drawn about the impact of the interruption. Pre-specification of the statistical method is encouraged, and naive conclusions based on statistical significance should be avoided.

## Supplementary Information

**Additional file 1.** Analysis code

**Additional file 2.** List of the studies that contributed data via publication, email or digital extraction

**Additional file 3: Appendix 1**: Interrupted time series with a transition period. **Appendix 2**: Difference in level and slope change by length of time series. **Appendix 3**: Standardising the direction of effect. **Appendix 4**: Detailed *p*-value comparisons.

## Data Availability

The datasets used and analysed during the current study are available from the corresponding author on request.

## Abbreviations

ARIMA: Autoregressive Integrated Moving Average
d.f.: Degrees of freedom
ICC: Intra-correlation coefficient
IQR: Inter-quartile range
ITS: Interrupted Time Series
LOESS: Local Regression
OLS: Ordinary Least Squares
NW: Newey-West
PW: Prais-Winsten
REML: Restricted Maximum Likelihood
REML-Satt: Restricted Maximum Likelihood with the small sample Satterthwaite approximation
RMSE: Root Mean Square Error
SE: Standard Error
