# Supplementary material for: Comparison of six statistical methods for interrupted time series studies: empirical evaluation of 190 published series
Source: BMC Med Res Methodol. 2021 Jun 26;21:134. doi: 10.1186/s12874-021-01306-w (PMC8235830; doi:10.1186/s12874-021-01306-w)
Supplement: Supplementary file 1 — Additional file 1. Analysis code [file 12874_2021_1306_MOESM1_ESM.docx]

# Additional File 1: analysis code

The following sections contain Stata 15 do files that analyse the data sets used in the study “Comparison of six statistical methods for interrupted time series studies: empirical evaluation of 190 published series”

Simon L Turner^1^, Amalia Karahalios^1^, Andrew B Forbes^1^, Monica Taljaard^2,3^, Jeremy M Grimshaw^2,3,4^, Joanne E McKenzie^1*^

^1^School of Public Health and Preventive Medicine, Monash University, Melbourne, Victoria, Australia.

^2^Clinical Epidemiology Program, Ottawa Hospital Research Institute, Ottawa, Ontario, Canada. 1053 Carling Ave, Ottawa.

^3^School of Epidemiology and Public Health, University of Ottawa, Ottawa, Ontario, Canada. 600 Peter Morand Crescent, Ottawa, Ontario K1G 5Z3.

^4^Department of Medicine, University of Ottawa, Ottawa, Ontario, Canada. Roger Guindon Hall, 451 Smyth Rd.

The following do files are required:

| Name of file | Purpose |
| --- | --- |
| 001_master_empirical_evaluation.do | Contains instructions on the running of the analysis and calls the subsequent do files |
| empirical_all_methods_published.do | Analyses each data series using a range of statistical methods and saves the results of each method in a separate .dta file |
| combine_methods_published.do | Combines the results from each statistical method into a single .dta file |
| get_wanted_empirical_estimates_published.do | The final data required does not include segments such as those used for transition periods, this do file selects the effect estimates used for the final summaries |

- 1. Data file 1: 001_master_empirical_evaluation.do

////////////////////////////////////////////////////////////////////////////////

// Master Empirical Evaluation final file

// Simon Turner

// For the study:

// "Comparison of six statistical methods for interrupted time series studies: empirical evaluation of 190 published series"

// Turner SL, Karahalios A, Forbes AB, Taljaard M, Grimshaw JM, McKenzie JE.

//

// This do file calls multiple other do files to:

//

// analyse each dataset using multiple statistical methods (empirical_all_methods_published.do)

// save level changes, slope changes, SEs, p-values and autocorrelation estimates

// combine all the results from the different methods (combine_methods_published.do)

// and choose the estimate wanted for analysis (get_wanted_empirical_estimates_published.do)

// the final file used for the manuscript (including graphs) is empirical_estimates.dta

//

// required data are found in the two excel files:

// STurner_Empirical_study_information.xls

// which contains information about the studies

// also

// STurner_Empirical_time_series.xls

// which contains the actual time series data

//

// further details about the variables can be found in:

// STurner_Empirical_Data_Dictionary.xls

////////////////////////////////////////////////////////////////////////////////

// firstly set the working directory...

global dir "<set directory>"

cd "$dir"

version 15

////////////////////////////////////////////////////////////////////////////////

// analyse the datasets

// now use different methods and capture the outputs

do ${dir}empirical_all_methods_published

// combine the results from the different methods

do ${dir}combine_methods_published

// for the empirical study we only want to use a subset of the total datasets

// this next section obtains the wanted datasets

do ${dir}get_wanted_empirical_estimates_published

////////////////////////////////////////////////////////////////////////////////

// The final file we can use for graphing, tables etc. is called:

// empirical_estimates_published.dta

////////////////////////////////////////////////////////////////////////////////

- 1. Data file 2: empirical_all_methods_published.do

////////////////////////////////////////////////////////////////////////////////

// This is the analysis file for the empirical study:

// "Comparison of six statistical methods for interrupted time series studies: empirical evaluation of 190 published series"

// loads the datasets,

// analyses each using a variety of statistical methods

// finally it saves the analysed data in "estimates_published_`model_type'"

// where `model_type' is one of the statistical methods used

// load the data that is ready for analysis

import excel "STurner_Empirical_time_series.xls", sheet("Sheet1") firstrow clear

// ensure that there is a proper study id

levelsof series_id, local(series_ids)

// there are several alternatives for scaling

// no scaling (just leave this blank)

// scaling by the rmse for the first segment only, use "rmse_on"

// or scaling by the rmse of the whole series, use "rmse_full"

// some of the datasets are very short (three points in pre-series) so first segment scaling does not work

// scaling by the full rmse was used for the analysis

local rmse_full = "rmse_full"

////////////////////////////////////////////////////////////////////////////////

// this short program estimates autocorrelation from the residuals after a

// simple linear regression

// this was not used in the analysis, but served as an interesting reference

cap program drop find_rho

program find_rho

syntax varlist [if]

marksample touse

quietly regress `varlist' l.`varlist' if `touse' , nocons

local rho=_b[L.`varlist']

scalar rho = `rho'

end

cap program drop find_rho_wrap

program find_rho_wrap

syntax varlist [if]

marksample touse

tempvar resid

regress `varlist' if `touse'

predict double `resid', resid

find_rho `resid' if `touse'

end

////////////////////////////////////////////////////////////////////////////////

// we investigated a range of statistical methods

// this string links to the sections below to identify which methods

// are going to be used here

// regress - OLS

// newey - OLS with newey-west standard errors

// prais - prais-winsten with an iterative search

// prais_raw - simple prais-winsten

// corc - cochrane-orcutt

// mixed - REML

// mixed_satt - REML with the Satterthwaite small series adjustment

// arima - ARIMA with lag-1

// for the final study we restricted these to the following...

local model_types "regress newey prais_raw mixed mixed_satt arima"

// loop over each statistical method

foreach model_type of local model_types {

// set the end file name

local save_name "estimates_published_`model_type'.dta"

// set up the temporary file used to store the data

// we are saving data per segment

// the level change and slope change with CIs, SEs and p-values

// autocorrelation estimates, degrees of freedom etc.

tempname post_values_`model_type'

postfile `post_values_`model_type'' series_id ///

str20 model_type ///

segment analysis_autocorr analysis_effects segment_num_points total_num_points rmse ///

level level_ll level_ul level_se level_p ///

slope slope_ll slope_ul slope_se slope_p ///

rho_est rho_cil rho_ciu ///

num_iterations error_code converged ///

lincom_level_dof lincom_slope_dof ///

using "`save_name'" , replace

// now for each of the data sets...

foreach series_id of local series_ids {

// going to just use one at a time

preserve

keep if series_id == `series_id'

local series_id = series_id[1]

display "working through `series_id'"

////////////////////////////////////////////////////////////////////////////

// set for program

*keep outcome time segment segment_in_analysis

sort time

drop if time == .

////////////////////////////////////////////////////////////////////////////////

// find times programatically

// this goes through and works out the timing of each segment

summ segment

local num_segments = r(max)

local min_seg_num = r(min)

if `min_seg_num' != 0 {

replace segment = segment - `min_seg_num'

}

summ segment

local num_segments = r(max)

local min_seg_num = r(min)

forvalues segment = 0/`num_segments' {

summ time if segment == `segment'

local time_`segment'_start = r(min)

local time_`segment'_end = r(max)

display "segment `segment' goes from `time_`segment'_start' to `time_`segment'_end'"

}

////////////////////////////////////////////////////////////////////////////////

// extra variables for analysis

// generate variables to indicate time of intervention

forvalues segment = 0/`num_segments' {

gen intervention_`segment' = 0

replace intervention_`segment' = 1 if segment >= `segment'

gen level_change_`segment' = intervention_`segment'

gen slope_change_`segment' = (time-`time_`segment'_start')*level_change_`segment'

}

////////////////////////////////////////////////////////////////////////////////

// model

////////////////////////////////////////////////////////////////////////////////

// create the variables to use (the various level and slope changes)

// starting with level_0 and slope_0, then incrementing for each segment

// e.g. regular segmented regression will have

// level_0 (intercept) slope_0 (pre-interruption slope) level_1 (level change at interruption) slope_1 (slope change post interruption)

local variables = ""

forvalues segment = 0/`num_segments' {

local variables = "`variables'" + " level_change_`segment' slope_change_`segment'"

}

display "variables: `variables'"

display "study: `year' number: `number_in_year' multiple: `multiple' data_type: `data_type'"

// first find rmse from all segments (for scaling)

regress outcome `variables', nocons

local rmse = e(rmse)

local total_num_points = e(N)

///////////////////////////////////////////

// now apply the correct statistical method according to model_type...

tsset time

local rho_est = .

local rho_est_cil = .

local rho_est_ciu = .

local model_error = 0

if "`model_type'" == "regress" { // basic OLS regression

regress outcome `variables', nocons

matrix local_results = r(table)

local rho_est = 0

local num_iterations = 1

local error_code = _rc

local converged = 1

} // end regress check

else if "`model_type'" == "newey" { // OLS regression with newey-west standard errors

newey outcome `variables', nocons lag(1) force // need force option to ignore missing values otherwise get time not equally spaced errors

matrix local_results = r(table)

local varlist = "outcome `variables'"

quietly find_rho_wrap `varlist'

local rho_est = rho

local num_iterations = 1

local error_code = _rc

local converged = 1

newey outcome `variables', nocons lag(1) force // need force option to ignore missing values otherwise get time not equally spaced errors

} // end newey check

else if "`model_type'" == "prais" { // Prais-Winsten with iterative search

cap prais outcome `variables', nocons ssesearch

if _rc != 0 {

local model_error = _rc

matrix local_results = J(6,5,.)

local rho_est = .

local converged = .

local num_iterations = e(ic)

local error_code = _rc

}

else {

matrix local_results = r(table)

local rho_est = e(rho)

local num_iterations = e(ic)

local error_code = _rc

local converged = 1

}

} // end prais check

else if "`model_type'" == "prais_raw" { // standard Prais-Winsten

cap prais outcome `variables', nocons

if _rc != 0 {

local model_error = _rc

matrix local_results = J(6,5,.)

local rho_est = .

local converged = .

local num_iterations = e(ic)

local error_code = _rc

}

else {

matrix local_results = r(table)

local rho_est = e(rho)

local num_iterations = e(ic)

local error_code = _rc

local converged = 1

}

} // end prais_raw check

else if "`model_type'" == "corc" { // Cochrane-Orcutt

cap prais outcome `variables', nocons corc ssesearch

if _rc != 0 {

local model_error = _rc

matrix local_results = J(6,5,.)

local rho_est = .

local converged = .

local num_iterations = e(ic)

local error_code = _rc

}

else {

matrix local_results = r(table)

local rho_est = e(rho)

local num_iterations = e(ic)

local error_code = _rc

local converged = 1

}

} // end corc check

else if "`model_type'" == "mixed" { // REML with maximum iterations set to 1000 to stop really long non-convergence

cap mixed outcome `variables', nocons res(ar 1, t(time)) var reml iter(1000)

if _rc != 0 {

local model_error = _rc

matrix local_results = J(6,5,.)

local rho_est = .

local converged = e(converged)

local num_iterations = e(ic)

local error_code = _rc

}

else {

matrix local_results = r(table)

local num_cols = colsof(local_results)

local rho_est = tanh(local_results[1,`num_cols'])

local rho_est_cil = tanh(local_results[5,`num_cols'])

local rho_est_ciu = tanh(local_results[6,`num_cols'])

local converged = e(converged)

local num_iterations = e(ic)

local error_code = _rc

}

} // end mixed check

else if "`model_type'" == "mixed_kr" { // REML with KR adjustment

cap mixed outcome `variables', nocons res(ar 1, t(time)) var reml iter(1000) dfmethod(kr)

if _rc != 0 {

local model_error = _rc

matrix local_results = J(6,5,.)

local rho_est = .

local converged = e(converged)

local num_iterations = e(ic)

local error_code = _rc

}

else {

matrix local_results = r(table)

local num_cols = colsof(local_results)

local rho_est = tanh(local_results[1,`num_cols'])

local rho_est_cil = tanh(local_results[5,`num_cols'])

local rho_est_ciu = tanh(local_results[6,`num_cols'])

local converged = e(converged)

local num_iterations = e(ic)

local error_code = _rc

mat dfs = e(df)

}

} // end mixed_kr check

else if "`model_type'" == "mixed_satt" { // REML with Satt adjustment

cap mixed outcome `variables', nocons res(ar 1, t(time)) var reml iter(1000) dfmethod(satt)

if _rc != 0 {

local model_error = _rc

matrix local_results = J(6,5,.)

local rho_est = .

local converged = e(converged)

local num_iterations = e(ic)

local error_code = _rc

}

else {

display "`model_type' model ran"

matrix local_results = r(table)

local num_cols = colsof(local_results)

local rho_est = tanh(local_results[1,`num_cols'])

local rho_est_cil = tanh(local_results[5,`num_cols'])

local rho_est_ciu = tanh(local_results[6,`num_cols'])

local converged = e(converged)

local num_iterations = e(ic)

local error_code = _rc

mat dfs = e(df)

}

} // end mixed_satt check

else if "`model_type'" == "arima" {

cap arima outcome `variables', nocons collinear ar(1) iter(1000)

if _rc != 0 {

// new section for error catch includes a "slow down" as for some reason

// Stata sometimes crashes if it went too quickly here (Stata 15.0)

local model_error = _rc

local iteration = 1

while `iteration' < 10 & _rc != 0 {

cap matrix local_results = J(6,5,.)

local rho_est = .

local converged = e(converged)

local num_iterations = e(ic)

local error_code = _rc

sleep 10

local iteration = `iteration' + 1

}

}

else {

matrix local_results = r(table)

local num_cols = colsof(local_results)

local rho_col = `num_cols' - 1

local rho_est = (local_results[1,`rho_col'])

local rho_est_cil = (local_results[5,`rho_col'])

local rho_est_ciu = (local_results[6,`rho_col'])

local converged = e(converged)

local num_iterations = e(ic)

local error_code = _rc

}

} // end arima check

else {

display "Model type `model_type' is not recognised"

stop

}

display "study: `year' number: `number_in_year' multiple: `multiple' data_type: `data_type' model `model_type' ran with error code `model_error'"

// if there was an error just set the output to missing values

if `model_error' != 0 {

forvalues segment = 0/`num_segments' {

local level_change_counter_`segment' = .

local level_change_counter_`segment'_ll = .

local level_change_counter_`segment'_ul = .

local level_change_counter_`segment'_se = .

local level_change_counter_`segment'_p = .

local slope_change_counter_`segment' = .

local slope_change_counter_`segment'_ll = .

local slope_change_counter_`segment'_ul = .

local slope_change_counter_`segment'_se = .

local slope_change_counter_`segment'_p = .

}

}

else {

// use lincom to find the CIs that go with the various level and slope changes

// predict the estimates to find the counterfactual, level change etc.

// base estimates of level change compared to first segment

predict estimates

gen counterfactual = _b[level_change_0] + _b[slope_change_0]*slope_change_0

// if satterthwaite is used need to add ",small" to the options after lincom

if "`model_type'" == "mixed_satt" {

local small " , small"

}

else {

local small ""

}

////////////////////////////////////////////////////////////////////////////////

// level changes from counterfactual

// for each segment...

forvalues segment = 0/`num_segments' {

// for the first segment just initialise everything

if `segment' == 0 {

local level_change_counter_`segment' = .

local level_change_counter_`segment'_ll = .

local level_change_counter_`segment'_ul = .

local level_change_counter_`segment'_se = .

local level_change_counter_`segment'_p = .

}

else {

// for each subsequent segment

// set up a local macro that holds the names of all the lincom variables we want

// this is going to be

// level_change_1 for the first level change

// and then adding on the subsequent level and slope change values for any subsequent segments

forvalues segment_sub = 1/`segment' {

if `segment_sub' == 1 {

local L`segment' = `" level_change_`segment_sub' "'

}

else {

local segment_multiplier = slope_change_`=`segment_sub'-1'[`time_`segment'_start']

local L`segment' = `" `L`segment'' "' + `" + slope_change_`=`segment_sub'-1'*`segment_multiplier' + level_change_`segment_sub' "'

}

*display "L`segment' `L`segment''"

}

// now lincom those variables (adding the small option if using REML-Satt)

lincom `L`segment'' `small'

// lincom gives slightly different responses in different situations...

if "`model_type'" == "arima" | "`model_type'" == "mixed" {

local lincom_level_dof = e(N) - e(df_m)

}

else {

local lincom_level_dof = r(df)

}

*return list

display "level lincom dof = `lincom_level_dof'"

// we are using a cut-off of 2 for the degrees of freedom for the REML-Satt method

if `lincom_level_dof' < 2 & "`model_type'" == "mixed_satt" {

lincom `L`segment'', df(2)

}

// now save those values

local level_change_counter_`segment' = r(estimate)

local level_change_counter_`segment'_ll = r(lb)

local level_change_counter_`segment'_ul = r(ub)

local level_change_counter_`segment'_se = r(se)

local level_change_counter_`segment'_p = r(p)

} // end if loop

} // end segment loop

////////////////////////////////////////////////////////////////////////////////

// slope changes from counterfactual

// this runs as the level change above, but for slope change values instead

forvalues segment = 0/`num_segments' {

if `segment' == 0 {

local slope_change_counter_`segment' = .

local slope_change_counter_`segment'_ll = .

local slope_change_counter_`segment'_ul = .

local slope_change_counter_`segment'_se = .

local slope_change_counter_`segment'_p = .

}

else {

forvalues segment_sub = 1/`segment' {

if `segment_sub' == 1 {

local SC_`segment' = "slope_change_`segment_sub'"

}

else {

local SC_`segment' = "`SC_`segment''" + " + slope_change_`segment_sub'"

}

}

lincom `SC_`segment'' `small'

if "`model_type'" == "arima" | "`model_type'" == "mixed" {

local lincom_slope_dof = e(N) - e(df_m)

}

else {

local lincom_slope_dof = r(df)

}

*return list

display "slope lincom dof = `lincom_slope_dof'"

if `lincom_slope_dof' < 2 & "`model_type'" == "mixed_satt" {

lincom `SC_`segment'', df(2)

}

local slope_change_counter_`segment' = r(estimate)

local slope_change_counter_`segment'_ll = r(lb)

local slope_change_counter_`segment'_ul = r(ub)

local slope_change_counter_`segment'_se = r(se)

local slope_change_counter_`segment'_p = r(p)

} // end if loop

} // end segment loop

} // end model error check.

////////////////////////////////////////////////////////////////////////////////

// postvalues

// finally put the values into the post file

forvalues segment = 0/`num_segments' {

local analysis_autocorr = analysis_autocorr[`time_`segment'_start']

// in the excel file the segments begin with 1, in this file we set to zero

// therefore we need to similarly reduce the analysis effects by 1 so they all match up

local analysis_effects = analysis_effects[`time_`segment'_start'] - 1

summ time if segment == `segment'

local segment_num_points = r(N)

// scaled adjustment by rmse

local level_change_counter_`segment' = `level_change_counter_`segment''/`rmse'

local level_change_counter_`segment'_ll = `level_change_counter_`segment'_ll'/`rmse'

local level_change_counter_`segment'_ul = `level_change_counter_`segment'_ul'/`rmse'

local level_change_counter_`segment'_se = `level_change_counter_`segment'_se'/`rmse'

local slope_change_counter_`segment' = `slope_change_counter_`segment''/`rmse'

local slope_change_counter_`segment'_ll = `slope_change_counter_`segment'_ll'/`rmse'

local slope_change_counter_`segment'_ul = `slope_change_counter_`segment'_ul'/`rmse'

local slope_change_counter_`segment'_se = `slope_change_counter_`segment'_se'/`rmse'

post `post_values_`model_type'' (`series_id') ///

("`model_type'") ///

(`segment') (`analysis_autocorr') (`analysis_effects') (`segment_num_points') (`total_num_points') (`rmse') ///

(`level_change_counter_`segment'') (`level_change_counter_`segment'_ll') (`level_change_counter_`segment'_ul') (`level_change_counter_`segment'_se') (`level_change_counter_`segment'_p') /// ///

(`slope_change_counter_`segment'') (`slope_change_counter_`segment'_ll') (`slope_change_counter_`segment'_ul') (`slope_change_counter_`segment'_se') (`slope_change_counter_`segment'_p') ///

(`rho_est') (`rho_est_cil') (`rho_est_ciu') ///

(`num_iterations') (`error_code') (`converged') (`lincom_level_dof') (`lincom_slope_dof')

} // end segment loop

restore

} // end study id loop

postclose `post_values_`model_type''

} // end of model type loop

- 1. Data file 3: combine_methods_published.do

////////////////////////////////////////////////////////////////////////////////

// This is the analysis file for the empirical study:

// "Comparison of six statistical methods for interrupted time series studies: empirical evaluation of 190 published series"

// this do file combines the results for all the different statistical methods

// the combined file is called estimates_published_all.dta

local model_types "regress newey prais_raw mixed mixed_satt arima"

local first = 0

foreach model of local model_types {

if `first' == 0 {

use "estimates_published_`model'.dta", clear

local first = 1

}

else {

append using "estimates_published_`model'.dta"

}

}

save estimates_published_all.dta, replace

- 1. Data file 4: get_wanted_empirical_estimates_published.do

////////////////////////////////////////////////////////////////////////////////

// This is the final do file for the empirical study:

// "Comparison of six statistical methods for interrupted time series studies: empirical evaluation of 190 published series"

// It selects the effect estimate wanted for final comparisons

// excluding segments that are not required (e.g. transition periods)

// It requires the data from STurner_Empirical_study_information which contains the desired segment number

// The final file "empirical_estimates_published.dta" is the output

// keep only the segments we need

keep if segment == analysis_effects

// tidy the file

sort series_id model_type segment

order series_id model_type segment segment_num_points total_num_points

// save the final data file

save empirical_estimates_published.dta, replace
