# Supplementary material for: Comparison of six statistical methods for interrupted time series studies: empirical evaluation of 190 published series
Source: BMC Med Res Methodol. 2021 Jun 26;21:134. doi: 10.1186/s12874-021-01306-w (PMC8235830; doi:10.1186/s12874-021-01306-w)
Supplement: Supplementary file 2 — Additional file 2. List of the studies that contributed data via publication, email or digital extraction [file 12874_2021_1306_MOESM2_ESM.docx]

# Additional File 2: List of the studies that contributed data via publication, email or digital extraction

The following file contains details of the type of data obtained from the 200 studies included in “Comparison of six statistical methods for interrupted time series studies: empirical evaluation of 190 published series”

Simon L Turner^1^, Amalia Karahalios^1^, Andrew B Forbes^1^, Monica Taljaard^2,3^, Jeremy M Grimshaw^2,3,4^, Joanne E McKenzie^1*^

^1^School of Public Health and Preventive Medicine, Monash University, Melbourne, Victoria, Australia.

^2^Clinical Epidemiology Program, Ottawa Hospital Research Institute, Ottawa, Ontario, Canada. 1053 Carling Ave, Ottawa.

^3^School of Epidemiology and Public Health, University of Ottawa, Ottawa, Ontario, Canada. 600 Peter Morand Crescent, Ottawa, Ontario K1G 5Z3.

^4^Department of Medicine, University of Ottawa, Ottawa, Ontario, Canada. Roger Guindon Hall, 451 Smyth Rd.

The following is a list of the studies that contributed data via publication, email or digital data extraction for the empirical evaluation study.

We wish to thank all of the authors who generously contributed datasets for this study.

| Study | Data via publication | Data via email | Data Extracted |
| --- | --- | --- | --- |
| Abegaz T, Berhane Y, Worku A, et al. Effectiveness of an improved road safety policy in Ethiopia: an interrupted time series study. BMC Public Health 2014;14(1) doi: 10.1186/1471-2458-14-539 | No | No | No |
| Adams AS, Soumerai SB, Zhang F, et al. Effects of Eliminating Drug Caps on Racial Differences in Antidepressant Use Among Dual Enrollees With Diabetes and Depression. Clinical Therapeutics 2015;37(3):597-609. doi: 10.1016/j.clinthera.2014.12.011 | No | No | Yes |
| Aiken AM, Wanyoro AK, Mwangi J, et al. Changing Use of Surgical Antibiotic Prophylaxis in Thika Hospital, Kenya: A Quality Improvement Intervention with an Interrupted Time Series Design. PLoS ONE 2013;8(11):e78942. doi: 10.1371/journal.pone.0078942 | No | No | Yes |
| Akhtar S, Ziyab AH. Impact of the Penalty Points System on Severe Road Traffic Injuries in Kuwait. Traffic Injury Prevention 2013;14(7):743-48. doi: 10.1080/15389588.2012.749466 | No | No | Yes |
| Alexandridis AA, McCort A, Ringwalt CL, et al. A statewide evaluation of seven strategies to reduce opioid overdose in North Carolina. Injury Prevention 2017;24(1):48-54. doi: 10.1136/injuryprev-2017-042396 | No | No | Yes |
| Alpert HR, Carpenter D, Connolly GN. Tobacco industry response to a ban on lights descriptors on cigarette packaging and population outcomes. Tobacco Control 2017;27(4):390-98. doi: 10.1136/tobaccocontrol-2017-053683 | Yes | N/A | Yes |
| Andersen SE, Knudsen JD. A managed multidisciplinary programme on multi-resistant Klebsiella pneumoniaein a Danish university hospital. BMJ Quality & Safety 2013;22(11):907-15. doi: 10.1136/bmjqs-2012-001791 | No | No | Yes |
| Andrade AL, Minamisava R, Policena G, et al. Evaluating the impact of PCV-10 on invasive pneumococcal disease in Brazil: A time-series analysis. Human Vaccines & Immunotherapeutics 2016;12(2):285-92. doi: 10.1080/21645515.2015.1117713 | No | Yes | No |
| Armah G, Pringle K, Enweronu-Laryea CC, et al. Impact and Effectiveness of Monovalent Rotavirus Vaccine Against Severe Rotavirus Diarrhea in Ghana. Clinical Infectious Diseases 2016;62(suppl 2):S200-S07. doi: 10.1093/cid/ciw014 | No | No | Yes |
| Barber C, Gagnon D, Fonda J, et al. Assessing the impact of prescribing directives on opioid prescribing practices among Veterans Health Administration providers. Pharmacoepidemiology and Drug Safety 2016;26(1):40-46. doi: 10.1002/pds.4066 | No | No | Yes |
| Barocas DA, Mallin K, Graves AJ, et al. Effect of the USPSTF Grade D Recommendation against Screening for Prostate Cancer on Incident Prostate Cancer Diagnoses in the United States. Journal of Urology 2015;194(6):1587-93. doi: 10.1016/j.juro.2015.06.075 | No | No | Yes |
| Baskerville NB, Brown KS, Nguyen NC, et al. Impact of Canadian tobacco packaging policy on use of a toll-free quit-smoking line: an interrupted time-series analysis. CMAJ Open 2016;4(1):E59-E65. doi: 10.9778/cmajo.20150104 | No | No | Yes |
| Been JV, Mackay DF, Millett C, et al. Impact of smoke-free legislation on perinatal and infant mortality: a national quasi-experimental study. Scientific Reports 2015;5(1) doi: 10.1038/srep13020 | No | No | No |
| Been JV, Szatkowski L, van Staa T-P, et al. Smoke-free legislation and the incidence of paediatric respiratory infections and wheezing/asthma: interrupted time series analyses in the four UK nations. Scientific Reports 2015;5(1) doi: 10.1038/srep15246 | No | No | No |
| Bell S, Davey P, Nathwani D, et al. Risk of AKI with Gentamicin as Surgical Prophylaxis. Journal of the American Society of Nephrology 2014;25(11):2625-32. doi: 10.1681/asn.2014010035 | No | Yes | Yes |
| Bendzsak AM, Baxter NN, Darling GE, et al. Regionalization and Outcomes of Lung Cancer Surgery in Ontario, Canada. Journal of Clinical Oncology 2017;35(24):2772-80. doi: 10.1200/jco.2016.69.8076 | No | No | Yes |
| Berkowitz SA, Percac-Lima S, Ashburner JM, et al. Building Equity Improvement into Quality Improvement: Reducing Socioeconomic Disparities in Colorectal Cancer Screening as Part of Population Health Management. Journal of General Internal Medicine 2015;30(7):942-49. doi: 10.1007/s11606-015-3227-4 | No | No | Yes |
| Bernat DH, Maldonado-Molina M, Hyland A, et al. Effects of Smoke-Free Laws on Alcohol-Related Car Crashes in California and New York: Time Series Analyses From 1982 to 2008. American Journal of Public Health 2013;103(2):214-20. doi: 10.2105/ajph.2012.300906 | No | No | No |
| Blais E, Carnis L. Improving the safety effect of speed camera programs through innovations: Evidence from the French experience. Journal of Safety Research 2015;55:135-45. doi: 10.1016/j.jsr.2015.08.007 | No | No | No |
| Bobo WV, Epstein RA, Hayes RM, et al. The effect of regulatory advisories on maternal antidepressant prescribing, 1995–2007: an interrupted time series study of 228,876 pregnancies. Archives of Women's Mental Health 2013;17(1):17-26. doi: 10.1007/s00737-013-0383-6 | No | No | Yes |
| Boden DG, Agarwal A, Hussain T, et al. Lowering levels of bed occupancy is associated with decreased inhospital mortality and improved performance on the 4-hour target in a UK District General Hospital. Emergency Medicine Journal 2015;33(2):85-90. doi: 10.1136/emermed-2014-204479 | No | No | Yes |
| Boel J, Andreasen V, Jarløv JO, et al. Impact of antibiotic restriction on resistance levels ofEscherichia coli: a controlled interrupted time series study of a hospital-wide antibiotic stewardship programme. Journal of Antimicrobial Chemotherapy 2016;71(7):2047-51. doi: 10.1093/jac/dkw055 | No | Yes | Yes |
| Bonander C, Nilson F, Andersson R. The effect of the Swedish bicycle helmet law for children: An interrupted time series study. Journal of Safety Research 2014;51:15-22. doi: 10.1016/j.jsr.2014.07.001 | No | Yes | Yes |
| Borde JP, Kern WV, Hug M, et al. Implementation of an intensified antibiotic stewardship programme targeting third-generation cephalosporin and fluoroquinolone use in an emergency medicine department. Emergency Medicine Journal 2014;32(7):509-15. doi: 10.1136/emermed-2014-204067 | No | No | Yes |
| Bowden JA, Dono J, John DL, et al. What happens when the price of a tobacco retailer licence increases? Tobacco Control 2013;23(2):178-80. doi: 10.1136/tobaccocontrol-2012-050615 | No | No | Yes |
| Bozorgmehr K, Razum O. Effect of Restricting Access to Health Care on Health Expenditures among Asylum-Seekers and Refugees: A Quasi-Experimental Study in Germany, 1994–2013. PLOS ONE 2015;10(7):e0131483. doi: 10.1371/journal.pone.0131483 | No | No | No |
| Branas CC, Kastanaki AE, Michalodimitrakis M, et al. The impact of economic austerity and prosperity events on suicide in Greece: a 30-year interrupted time-series analysis. BMJ Open 2015;5(1):e005619-e19. doi: 10.1136/bmjopen-2014-005619 | No | No | No |
| Bugden S, Friesen KJ, Falk J. Voluntary warnings and the limits of good prescribing behavior: the case for de-adoption of meperidine. Journal of Pain Research 2015:879. doi: 10.2147/jpr.s96625 | No | No | Yes |
| Burke LK, Brown CP, Johnson TM. Historical Data Analysis of Hospital Discharges Related to the Amerithrax Attack in Florida. Perspect Health Inf Manag 2016;13(Fall):1c-1c. | No | No | No |
| Busch SH, McGinty EE, Stuart EA, et al. Was federal parity associated with changes in Out-of-network mental health care use and spending? BMC Health Services Research 2017;17(1) doi: 10.1186/s12913-017-2261-9 | No | No | Yes |
| Cairns KA, Jenney AWJ, Abbott IJ, et al. Prescribing trends before and after implementation of an antimicrobial stewardship program. The Medical Journal of Australia 2013;198(5):262-66. doi: 10.5694/mja12.11683 | No | No | Yes |
| Carracedo-Martínez E, Pia-Morandeira A, Figueiras A. Trends in celecoxib and etoricoxib prescribing following removal of prior authorization requirement in Spain. Journal of Clinical Pharmacy and Therapeutics 2016;42(2):185-88. doi: 10.1111/jcpt.12490 | No | Yes | Yes |
| Cecil E, Bottle A, Sharland M, et al. Impact of UK Primary Care Policy Reforms on Short-Stay Unplanned Hospital Admissions for Children With Primary Care-Sensitive Conditions. The Annals of Family Medicine 2015;13(3):214-20. doi: 10.1370/afm.1786 | No | No | No |
| Chandran A, Pérez-Núñez R, Bachani AM, et al. Early Impact of a National Multi-Faceted Road Safety Intervention Program in Mexico: Results of a Time-Series Analysis. PLoS ONE 2014;9(1):e87482. doi: 10.1371/journal.pone.0087482 | No | Yes | Yes |
| Chang C-H, Lin J-W, Wu L-C, et al. National Antiviral Treatment Program and the Incidence of Hepatocellular Carcinoma and Associated Mortality in Taiwan. Medical Care 2013;51(10):908-13. doi: 10.1097/mlr.0b013e3182a502ba | No | No | No |
| Chen IL, Lee C-H, Su L-H, et al. Effects of implementation of an online comprehensive antimicrobial-stewardship program in ICUs: A longitudinal study. Journal of Microbiology, Immunology and Infection 2018;51(1):55-63. doi: 10.1016/j.jmii.2016.06.007 | No | No | Yes |
| Cheng C-L, Chao P-H, Hsu JC-S, et al. Utilization patterns of Antihyperuricemic Agents Following Safety Announcement on Allopurinol and Benzbromarone by Taiwan Food and Drug Administration. Pharmacoepidemiology and Drug Safety 2013;23(3):309-13. doi: 10.1002/pds.3550 | No | No | Yes |
| Cheng J, Benassi P, de Oliveira C, et al. Impact of a mass media mental health campaign on psychiatric emergency department visits. Canadian Journal of Public Health 2016;107(3):e303-e11. doi: 10.17269/cjph.107.5265 | No | No | Yes |
| Chua K-P, Shrime MG, Conti RM. Effect of FDA Investigation on Opioid Prescribing to Children After Tonsillectomy/Adenoidectomy. Pediatrics 2017;140(6):e20171765. doi: 10.1542/peds.2017-1765 | No | No | Yes |
| Chung YK, Kim J-S, Lee SS, et al. Effect of daily chlorhexidine bathing on acquisition of carbapenem-resistant Acinetobacter baumannii (CRAB) in the medical intensive care unit with CRAB endemicity. American Journal of Infection Control 2015;43(11):1171-77. doi: 10.1016/j.ajic.2015.07.001 | No | No | Yes |
| Čižman M, Plankar Srovin T, Blagus R, et al. The long-term effects of restrictive interventions on consumption and costs of antibiotics. Journal of Global Antimicrobial Resistance 2015;3(1):31-35. doi: 10.1016/j.jgar.2014.11.004 | No | No | Yes |
| Corcoran P, Griffin E, Arensman E, et al. Impact of the economic recession and subsequent austerity on suicide and self-harm in Ireland: An interrupted time series analysis. International Journal of Epidemiology 2015;44(3):969-77. doi: 10.1093/ije/dyv058 | No | No | Yes |
| Cunningham JK, Liu L-M, Callaghan RC. Essential (“Precursor”) chemical control for heroin: Impact of acetic anhydride regulation on US heroin availability. Drug and Alcohol Dependence 2013;133(2):520-28. doi: 10.1016/j.drugalcdep.2013.07.014 | No | No | Yes |
| Damiani G, Federico B, Anselmi A, et al. The impact of Regional co-payment and National reimbursement criteria on statins use in Italy: an interrupted time-series analysis. BMC Health Services Research 2014;14(1) doi: 10.1186/1472-6963-14-6 | No | No | Yes |
| Denkel LA, Schwab F, Garten L, et al. Protective Effect of Dual-Strain Probiotics in Preterm Infants: A Multi-Center Time Series Analysis. PLOS ONE 2016;11(6):e0158136. doi: 10.1371/journal.pone.0158136 | No | Yes | Yes |
| Desai SP, Lu B, Szent-Gyorgyi LE, et al. Increasing pneumococcal vaccination for immunosuppressed patients: A cluster quality improvement trial. Arthritis & Rheumatism 2012;65(1):39-47. doi: 10.1002/art.37716 | No | No | Yes |
| Deslandes PN, Jenkins KSL, Haines KE, et al. A change in the trend in dosulepin usage following the introduction of a prescribing indicator but not after two national safety warnings. Journal of Clinical Pharmacy and Therapeutics 2016;41(2):224-28. doi: 10.1111/jcpt.12376 | No | Yes | Yes |
| Dicks KV, Lofgren E, Lewis SS, et al. A Multicenter Pragmatic Interrupted Time Series Analysis of Chlorhexidine Gluconate Bathing in Community Hospital Intensive Care Units. Infection Control & Hospital Epidemiology 2016;37(7):791-97. doi: 10.1017/ice.2016.23 | No | Yes | No |
| Dik J-WH, Hendrix R, Lo-Ten-Foe JR, et al. Automatic day-2 intervention by a multidisciplinary antimicrobial stewardship-team leads to multiple positive effects. Frontiers in Microbiology 2015;06 doi: 10.3389/fmicb.2015.00546 | No | No | Yes |
| DiMaggio C, Chen Q, Muennig PA, et al. Timing and effect of a safe routes to school program on child pedestrian injury risk during school travel hours: Bayesian changepoint and difference-in-differences analysis. Injury Epidemiology 2014;1(1) doi: 10.1186/s40621-014-0017-0 | No | No | Yes |
| Doernberg SB, Dudas V, Trivedi KK. Implementation of an antimicrobial stewardship program targeting residents with urinary tract infections in three community long-term care facilities: a quasi-experimental study using time-series analysis. Antimicrobial Resistance and Infection Control 2015;4(1) doi: 10.1186/s13756-015-0095-y | No | No | Yes |
| Dresden SM, Powell ES, Kang R, et al. Increased Emergency Department Use in Illinois After Implementation of the Patient Protection and Affordable Care Act. Annals of Emergency Medicine 2017;69(2):172-80. doi: 10.1016/j.annemergmed.2016.06.026 | No | No | Yes |
| Druetz T, Fregonese F, Bado A, et al. Abolishing Fees at Health Centers in the Context of Community Case Management of Malaria: What Effects on Treatment-Seeking Practices for Febrile Children in Rural Burkina Faso? PLOS ONE 2015;10(10):e0141306. doi: 10.1371/journal.pone.0141306 | No | No | No |
| Emmerick ICM, Campos MR, Luiza VL, et al. Retrospective interrupted time series examining hypertension and diabetes medicines usage following changes in patient cost sharing in the ‘Farmácia Popular’ programme in Brazil. BMJ Open 2017;7(11):e017308. doi: 10.1136/bmjopen-2017-017308 | No | No | Yes |
| Faryar KA, Freeman CL, Persaud AK, et al. The Effects of Kentucky's Comprehensive Opioid Legislation on Patients Presenting with Prescription Opioid or Heroin Abuse to One Urban Emergency Department. The Journal of Emergency Medicine 2017;53(6):805-14. doi: 10.1016/j.jemermed.2017.08.066 | No | No | Yes |
| Filippidis FT, Gerovasili V, Millett C, et al. Medium-term impact of the economic crisis on mortality, health-related behaviours and access to healthcare in Greece. Scientific Reports 2017;7(1) doi: 10.1038/srep46423 | Yes | N/A | No |
| Finnell KJ, John R, Thompson DM. 1% low-fat milk has perks!: An evaluation of a social marketing intervention. Preventive Medicine Reports 2017;5:144-49. doi: 10.1016/j.pmedr.2016.11.017 | No | No | Yes |
| Fisher D, Tambyah PA, Lin RTP, et al. Sustained meticillin-resistant Staphylococcus aureus control in a hyper-endemic tertiary acute care hospital with infrastructure challenges in Singapore. Journal of Hospital Infection 2013;85(2):141-48. doi: 10.1016/j.jhin.2013.07.005 | No | Yes | Yes |
| Flett KB, Ozonoff A, Graham DA, et al. Impact of Mandatory Public Reporting of Central Line–Associated Bloodstream Infections on Blood Culture and Antibiotic Utilization in Pediatric and Neonatal Intensive Care Units. Infection Control & Hospital Epidemiology 2015;36(8):878-85. doi: 10.1017/ice.2015.100 | No | No | Yes |
| Flynn D, Ford GA, Rodgers H, et al. A Time Series Evaluation of the FAST National Stroke Awareness Campaign in England. PLoS ONE 2014;9(8):e104289. doi: 10.1371/journal.pone.0104289 | No | No | Yes |
| Fournier P, Dumont A, Tourigny C, et al. The Free Caesareans Policy in Low-Income Settings: An Interrupted Time Series Analysis in Mali (2003–2012). PLoS ONE 2014;9(8):e105130. doi: 10.1371/journal.pone.0105130 | No | No | Yes |
| Gadzhanova SV, Roughead EE, Bartlett MJ. Improving cardiovascular disease management in Australia: NPS MedicineWise. The Medical Journal of Australia 2013;199(3):192-95. doi: 10.5694/mja12.11779 | No | No | Yes |
| Gale M, Muscatello DJ, Dinh M, et al. Alcopops, taxation and harm: a segmented time series analysis of emergency department presentations. BMC Public Health 2015;15(1) doi: 10.1186/s12889-015-1769-3 | No | No | Yes |
| Gallini A, Andrieu S, Donohue JM, et al. Trends in use of antipsychotics in elderly patients with dementia: Impact of national safety warnings. European Neuropsychopharmacology 2014;24(1):95-104. doi: 10.1016/j.euroneuro.2013.09.003 | No | No | Yes |
| Gamble J-M, Johnson JA, Majumdar SR, et al. Evaluating the introduction of a computerized prior-authorization system on the completeness of drug exposure data. Pharmacoepidemiology and Drug Safety 2013;22(5):551-55. doi: 10.1002/pds.3427 | No | No | Yes |
| Garnett M, Charyk Stewart T, Miller MR, et al. Did Amendments to the Ontario Highway Traffic Act in 2009-2010 Affect the Proportion of Alcohol-Related Motor Vehicle Collisions Seen at a Level I Trauma Centre over a 10-year Period? CJEM 2016;19(2):106-11. doi: 10.1017/cem.2016.343 | No | Yes | Yes |
| Gaudreau K, Sanford CJ, Cheverie C, et al. The Effect of a Smoking Ban on Hospitalization Rates for Cardiovascular and Respiratory Conditions in Prince Edward Island, Canada. PLoS ONE 2013;8(3):e56102. doi: 10.1371/journal.pone.0056102 | No | No | Yes |
| Gebrehiwot TG, San Sebastian M, Edin K, et al. The Health Extension Program and Its Association with Change in Utilization of Selected Maternal Health Services in Tigray Region, Ethiopia: A Segmented Linear Regression Analysis. PLOS ONE 2015;10(7):e0131195. doi: 10.1371/journal.pone.0131195 | Yes | N/A | Yes |
| Gefenaite G, Bijlsma M, Bos H, et al. Did introduction of pneumococcal vaccines in the Netherlands decrease the need for respiratory antibiotics in children? Analysis of 2002 to 2013 data. Eurosurveillance 2014;19(44):20948. doi: 10.2807/1560-7917.es2014.19.44.20948 | No | No | No |
| Gilbert C, Darlow B, Zin A, et al. Educating Neonatal Nurses in Brazil: A Before-and-After Study with Interrupted Time Series Analysis. Neonatology 2014;106(3):201-08. doi: 10.1159/000362532 | No | No | Yes |
| Glantz SA, Gibbs E. Changes in Ambulance Calls After Implementation of a Smoke-Free Law and Its Extension to Casinos. Circulation 2013;128(8):811-13. doi: 10.1161/circulationaha.113.003455 | No | No | Yes |
| Gobin M, Verlander N, Maurici C, et al. Do sexual health campaigns work? An outcome evaluation of a media campaign to increase chlamydia testing among young people aged 15–24 in England. BMC Public Health 2013;13(1) doi: 10.1186/1471-2458-13-484 | No | Yes | Yes |
| Godman B, Persson M, Miranda J, et al. Changes in the Utilization of Venlafaxine after the Introduction of Generics in Sweden. Applied Health Economics and Health Policy 2013;11(4):383-93. doi: 10.1007/s40258-013-0037-x | No | No | Yes |
| Godman B, Wettermark B, Miranda J, et al. Influence of multiple initiatives in Sweden to enhance ARB prescribing efficiency following generic losartan; findings and implications for other countries. International Journal of Clinical Practice 2013;67(9):853-62. doi: 10.1111/ijcp.12130 | No | No | Yes |
| Gold R, Nelson C, Cowburn S, et al. Feasibility and impact of implementing a private care system’s diabetes quality improvement intervention in the safety net: a cluster-randomized trial. Implementation Science 2015;10(1) doi: 10.1186/s13012-015-0259-4 | No | No | Yes |
| Graves AJ, Kozhimannil KB, Kleinman KP, et al. The Association between High-Deductible Health Plan Transition and Contraception and Birth Rates. Health Services Research 2015;51(1):187-204. doi: 10.1111/1475-6773.12326 | No | No | No |
| Guthrie B, Clark SA, Reynish EL, et al. Differential Impact of Two Risk Communications on Antipsychotic Prescribing to People with Dementia in Scotland: Segmented Regression Time Series Analysis 2001–2011. PLoS ONE 2013;8(7):e68976. doi: 10.1371/journal.pone.0068976 | No | Yes | Yes |
| Haas JP, Menz J, Dusza S, et al. Implementation and impact of ultraviolet environmental disinfection in an acute care setting. American Journal of Infection Control 2014;42(6):586-90. doi: 10.1016/j.ajic.2013.12.013 | No | No | Yes |
| Haggins A, Patrick S, Demonner S, et al. When Coverage Expands: Children's Health Insurance Program as a Natural Experiment in Use of Health Care Services. Academic Emergency Medicine 2013;20(10):1026-32. doi: 10.1111/acem.12236 | No | No | Yes |
| Halim S, Jiang H. The effect of Operation 24 Hours on reducing collision in the City of Edmonton. Accident Analysis & Prevention 2013;58:106-14. doi: 10.1016/j.aap.2013.04.031 | No | No | Yes |
| Hamilton I, Lloyd C, Bland JM, et al. The impact of assertive outreach teams on hospital admissions for psychosis: a time series analysis. Journal of Psychiatric and Mental Health Nursing 2015;22(7):484-90. doi: 10.1111/jpm.12239 | No | No | Yes |
| Hanatani T, Sai K, Tohkin M, et al. Evaluation of two Japanese regulatory actions using medical information databases: a ‘Dear Doctor’ letter to restrict oseltamivir use in teenagers, and label change caution against co-administration of omeprazole with clopidogrel. Journal of Clinical Pharmacy and Therapeutics 2014;39(4):361-67. doi: 10.1111/jcpt.12153 | No | No | Yes |
| Hansen BT, Østergaard SD, Sønderskov KM, et al. Increased Incidence Rate of Trauma- and Stressor-Related Disorders in Denmark After the September 11, 2001, Terrorist Attacks in the United States. American Journal of Epidemiology 2016;184(7):494-500. doi: 10.1093/aje/kww089 | No | Yes | No |
| Hansen BT, Sønderskov KM, Hageman I, et al. Daylight Savings Time Transitions and the Incidence Rate of Unipolar Depressive Episodes. Epidemiology 2017;28(3):346-53. doi: 10.1097/ede.0000000000000580 | Yes | N/A | Yes |
| Harper S, Bruckner TA. Did the Great Recession increase suicides in the USA? Evidence from an interrupted time-series analysis. Annals of Epidemiology 2017;27(7):409-14.e6. doi: 10.1016/j.annepidem.2017.05.017 | No | Yes | No |
| Hartung DM, Middleton L, Markwardt S, et al. Changes in Long-acting β-agonist Utilization After the FDA’s 2010 Drug Safety Communication. Clinical Therapeutics 2015;37(1):114-23.e1. doi: 10.1016/j.clinthera.2014.10.025 | No | No | Yes |
| Hassanian-Moghaddam H, Ghorbani F, Rahimi A, et al. Federation Internationale de Football Association (FIFA) 2014 World Cup Impact on Hospital-Treated Suicide Attempt (Overdose) in Tehran. Suicide and Life-Threatening Behavior 2017;48(3):367-75. doi: 10.1111/sltb.12359 | No | Yes | Yes |
| Hawton K, Bergen H, Geulayov G, et al. Impact of the recent recession on self-harm: Longitudinal ecological and patient-level investigation from the Multicentre Study of Self-harm in England. Journal of Affective Disorders 2016;191:132-38. doi: 10.1016/j.jad.2015.11.001 | No | No | Yes |
| Hawton K, Bergen H, Simkin S, et al. Long term effect of reduced pack sizes of paracetamol on poisoning deaths and liver transplant activity in England and Wales: interrupted time series analyses. BMJ 2013;346(feb07 1):f403-f03. doi: 10.1136/bmj.f403 | No | No | Yes |
| Hingwala J, Bhangoo S, Hiebert B, et al. Evaluating the Implementation Strategy for Estimated Glomerular Filtration Rate Reporting in Manitoba: The Effect on Referral Numbers, Wait Times, and Appropriateness of Consults. Canadian Journal of Kidney Health and Disease 2014;1:9. doi: 10.1186/2054-3581-1-9 | No | Yes | Yes |
| Høgli JU, Garcia BH, Skjold F, et al. An audit and feedback intervention study increased adherence to antibiotic prescribing guidelines at a Norwegian hospital. BMC Infectious Diseases 2016;16(1) doi: 10.1186/s12879-016-1426-1 | No | No | Yes |
| Honein-AbouHaidar GN, Rabeneck L, Paszat LF, et al. Evaluating the impact of public health initiatives on trends in fecal occult blood test participation in Ontario. BMC Cancer 2014;14(1) doi: 10.1186/1471-2407-14-537 | No | No | Yes |
| Horton DB, Gerhard T, Davidow A, et al. Impact of the black triangle label on prescribing of new drugs in the United Kingdom: lessons for the United States at a time of deregulation. Pharmacoepidemiology and Drug Safety 2017;26(11):1307-13. doi: 10.1002/pds.4304 | No | No | No |
| Hostenkamp G, Fischer KE, Borch-Johnsen K. Drug safety and the impact of drug warnings: An interrupted time series analysis of diabetes drug prescriptions in Germany and Denmark. Health Policy 2016;120(12):1404-11. doi: 10.1016/j.healthpol.2016.09.020 | No | No | No |
| Hsu JC, Cheng C-L, Ross-Degnan D, et al. Effects of safety warnings and risk management plan for Thiazolidinediones in Taiwan. Pharmacoepidemiology and Drug Safety 2015;24(10):1026-35. doi: 10.1002/pds.3834 | No | Yes | Yes |
| Hsu JC, Lu CY, Wagner AK, et al. Impacts of drug reimbursement reductions on utilization and expenditures of oral antidiabetic medications in Taiwan: An interrupted time series study. Health Policy 2014;116(2-3):196-205. doi: 10.1016/j.healthpol.2013.11.005 | No | Yes | Yes |
| Huitema BE, Van Houten R, Manal H. Time-series intervention analysis of pedestrian countdown timer effects. Accident Analysis & Prevention 2014;72:23-31. doi: 10.1016/j.aap.2014.05.025 | No | No | No |
| Humphreys DK, Gasparrini A, Wiebe DJ. Evaluating the Impact of Florida’s “Stand Your Ground” Self-defense Law on Homicide and Suicide by Firearm. JAMA Internal Medicine 2017;177(1):44. doi: 10.1001/jamainternmed.2016.6811 | No | Yes | Yes |
| Iams W, Heck J, Kapp M, et al. A Multidisciplinary Housestaff-Led Initiative to Safely Reduce Daily Laboratory Testing. Academic Medicine 2016;91(6):813-20. doi: 10.1097/acm.0000000000001149 | No | No | Yes |
| Jenkins TC, Knepper BC, Shihadeh K, et al. Long-Term Outcomes of an Antimicrobial Stewardship Program Implemented in a Hospital with Low Baseline Antibiotic Use. Infection Control & Hospital Epidemiology 2015;36(6):664-72. doi: 10.1017/ice.2015.41 | No | No | Yes |
| Jiao B, Kim S, Hagen J, et al. Cost-effectiveness of neighbourhood slow zones in New York City. Injury Prevention 2017;25(2):98-103. doi: 10.1136/injuryprev-2017-042499 | Yes | N/A | No |
| Johri M, Ridde V, Heinmüller R, et al. Estimation of maternal and child mortality one year after user-fee elimination: an impact evaluation and modelling study in Burkina Faso. Bulletin of the World Health Organization 2014;92(10):706-15. doi: 10.2471/blt.13.130609 | No | No | No |
| Katikireddi SV, Der G, Roberts C, et al. Has Childhood Smoking Reduced Following Smoke-Free Public Places Legislation? A Segmented Regression Analysis of Cross-Sectional UK School-Based Surveys. Nicotine & Tobacco Research 2016;18(7):1670-74. doi: 10.1093/ntr/ntw018 | Yes | N/A | Yes |
| Kesselheim AS, Donneyong M, Dal Pan GJ, et al. Changes in prescribing and healthcare resource utilization after FDA Drug Safety Communications involving zolpidem-containing medications. Pharmacoepidemiology and Drug Safety 2017;26(6):712-21. doi: 10.1002/pds.4215 | No | No | Yes |
| Kim B, Kim K, Lee J, et al. Impact of bacteremia prediction rule in CAP: Before and after study. The American Journal of Emergency Medicine 2018;36(5):758-62. doi: 10.1016/j.ajem.2017.10.005 | No | No | Yes |
| Kim J-S, Chung YK, Lee SS, et al. Effect of daily chlorhexidine bathing on the acquisition of methicillin-resistant Staphylococcus aureus in a medical intensive care unit with methicillin-resistant S aureus endemicity. American Journal of Infection Control 2016;44(12):1520-25. doi: 10.1016/j.ajic.2016.04.252 | No | No | Yes |
| Kim SH, Cho BL, Shin DW, et al. The Effect of Asthma Clinical Guideline for Adults on Inhaled Corticosteroids PrescriptionTrend: A Quasi-Experimental Study. Journal of Korean Medical Science 2015;30(8):1048. doi: 10.3346/jkms.2015.30.8.1048 | No | No | Yes |
| Kiran T, Wilton AS, Moineddin R, et al. Effect of Payment Incentives on Cancer Screening in Ontario Primary Care. The Annals of Family Medicine 2014;12(4):317-23. doi: 10.1370/afm.1664 | No | Yes | Yes |
| Kisely S, Crowe E, Lawrence D, et al. A time series analysis of presentations to Queensland health facilities for alcohol-related conditions, following the increase in ‘alcopops’ tax. Australasian Psychiatry 2013;21(4):383-88. doi: 10.1177/1039856213486307 | No | No | Yes |
| Klein EG, Forster JL, Toomey TL, et al. Did a local clean indoor air policy increase alcohol-related crime around bars and restaurants? Tobacco Control 2011;22(2):113-17. doi: 10.1136/tobaccocontrol-2011-050010 | No | No | No |
| Kolhatkar A, Cheng L, Chan FKI, et al. The impact of medication reviews by community pharmacists. Journal of the American Pharmacists Association 2016;56(5):513-20.e1. doi: 10.1016/j.japh.2016.05.002 | No | Yes | Yes |
| Kontopantelis E, Olier I, Planner C, et al. Primary care consultation rates among people with and without severe mental illness: a UK cohort study using the Clinical Practice Research Datalink. BMJ Open 2015;5(12):e008650. doi: 10.1136/bmjopen-2015-008650 | Yes | N/A | Yes |
| Kontopantelis E, Reeves D, Valderas JM, et al. Recorded quality of primary care for patients with diabetes in England before and after the introduction of a financial incentive scheme: a longitudinal observational study. BMJ Quality & Safety 2012;22(1):53-64. doi: 10.1136/bmjqs-2012-001033 | No | Yes | Yes |
| Kracalik I, Abdullayev R, Asadov K, et al. Changing Patterns of Human Anthrax in Azerbaijan during the Post-Soviet and Preemptive Livestock Vaccination Eras. PLoS Neglected Tropical Diseases 2014;8(7):e2985. doi: 10.1371/journal.pntd.0002985 | No | No | Yes |
| Kracalik IT, Abdullayev R, Asadov K, et al. Human Brucellosis Trends: Re-emergence and Prospects for Control Using a One Health Approach in Azerbaijan (1983-2009). Zoonoses and Public Health 2015;63(4):294-302. doi: 10.1111/zph.12229 | No | No | Yes |
| Kruik-Kollöffel WJ, van der Palen J, Kruik HJ, et al. Prescription behavior for gastroprotective drugs in new users as a result of communications regarding clopidogrel - proton pump inhibitor interaction. Pharmacology Research & Perspectives 2016;4(4):e00242. doi: 10.1002/prp2.242 | No | Yes | Yes |
| Larney S, Lai W, Dolan K, et al. Monitoring a Prison Opioid Treatment Program Over a Period of Change to Clinical Governance Arrangements, 2007–2013. Journal of Substance Abuse Treatment 2016;70:58-63. doi: 10.1016/j.jsat.2016.08.001 | No | No | Yes |
| Lavergne MR, Law MR, Peterson S, et al. Effect of incentive payments on chronic disease management and health services use in British Columbia, Canada: Interrupted time series analysis. Health Policy 2018;122(2):157-64. doi: 10.1016/j.healthpol.2017.11.001 | No | Yes | Yes |
| Lee KR, Bagga B, Arnold SR. Reduction of Broad-Spectrum Antimicrobial Use in a Tertiary Children’s Hospital Post Antimicrobial Stewardship Program Guideline Implementation*. Pediatric Critical Care Medicine 2016;17(3):187-93. doi: 10.1097/pcc.0000000000000615 | No | No | Yes |
| Lee TC, Frenette C, Jayaraman D, et al. Antibiotic Self-stewardship: Trainee-Led Structured Antibiotic Time-outs to Improve Antimicrobial Use. Annals of Internal Medicine 2014;161(10_Supplement):S53. doi: 10.7326/m13-3016 | No | No | Yes |
| Lee Y-J, Chen J-Z, Lin H-C, et al. Impact of active screening for methicillin-resistant Staphylococcus aureus (MRSA) and decolonization on MRSA infections, mortality and medical cost: a quasi-experimental study in surgical intensive care unit. Critical Care 2015;19(1) doi: 10.1186/s13054-015-0876-y | No | Yes | Yes |
| Li Z, Li M, Fink G, et al. User–fee–removal improves equity of children’s health care utilization and reduces families’ financial burden: evidence from Jamaica. Journal of Global Health 2017;7(1) doi: 10.7189/jogh.07.010502 | No | No | Yes |
| Lieberman DA, Polinski JM, Choudhry NK, et al. Unintended Consequences of a Medicaid Prescription Copayment Policy. Medical Care 2014;52(5):422-27. doi: 10.1097/mlr.0000000000000119 | No | No | Yes |
| Lin CM, Liao CM. Inpatient expenditures on alcohol-attributed diseases and alcohol tax policy: a nationwide analysis in Taiwan from 1996 to 2010. Public Health 2014;128(11):977-84. doi: 10.1016/j.puhe.2014.09.004 | No | Yes | Yes |
| Lopez Bernal J, Gasparrini A, Artundo C, et al. RE: The effect of the late 2000s financial crisis on suicides in Spain: an interrupted time-series analysis. The European Journal of Public Health 2014;24(2):183-84. doi: 10.1093/eurpub/ckt215 | No | No | Yes |
| López-Ruiz M, Martínez JM, Pérez K, et al. Impact of road safety interventions on traffic-related occupational injuries in Spain, 2004–2010. Accident Analysis & Prevention 2014;66:114-19. doi: 10.1016/j.aap.2014.01.012 | No | No | Yes |
| Lu CY, Zhang F, Lakoma MD, et al. Asthma Treatments and Mental Health Visits After a Food and Drug Administration Label Change for Leukotriene Inhibitors. Clinical Therapeutics 2015;37(6):1280-91. doi: 10.1016/j.clinthera.2015.03.027 | No | No | Yes |
| Ma T, Byrne PA, Haya M, et al. Working in tandem: The contribution of remedial programs and roadside licence suspensions to drinking and driving deterrence in Ontario. Accident Analysis & Prevention 2015;85:248-56. doi: 10.1016/j.aap.2015.09.017 | No | No | Yes |
| Maini R, Van den Bergh R, van Griensven J, et al. Picking up the bill - improving health-care utilisation in the Democratic Republic of Congo through user fee subsidisation: a before and after study. BMC Health Services Research 2014;14(1) doi: 10.1186/s12913-014-0504-6 | No | Yes | Yes |
| Martin CL, Aldridge PJ, Harris AM, et al. Opening a New Level II Trauma Center Near an Established Level I Trauma Center. Journal of Orthopaedic Trauma 2016;30(10):517-23. doi: 10.1097/bot.0000000000000640 | No | No | Yes |
| Marufu O, Desai N, Aldred D, et al. Analysis of interventions to reduce the incidence of Clostridium difficile infection at a London teaching hospital trust, 2003‒2011. Journal of Hospital Infection 2015;89(1):38-45. doi: 10.1016/j.jhin.2014.10.003 | No | No | Yes |
| Marwick CA, Guthrie B, Pringle JEC, et al. A multifaceted intervention to improve sepsis management in general hospital wards with evaluation using segmented regression of interrupted time series. BMJ Quality & Safety 2013;23(12):e2-e2. doi: 10.1136/bmjqs-2013-002176 | No | Yes | Yes |
| McAlister FA, Bakal JA, Kaul P, et al. Changes in Heart Failure Outcomes After a Province-Wide Change in Health Service Provision A Natural Experiment in Alberta, Canada. Circulation: Heart Failure 2013;6(1):76-82. doi: 10.1161/circheartfailure.112.971119 | No | No | No |
| McFarlane WR, Susser E, McCleary R, et al. Reduction in Incidence of Hospitalizations for Psychotic Episodes Through Early Identification and Intervention. Psychiatric Services 2014;65(10):1194-200. doi: 10.1176/appi.ps.201300336 | No | Yes | No |
| McKirdy A, Imbuldeniya AM. The clinical and cost effectiveness of a virtual fracture clinic service. Bone & Joint Research 2017;6(5):259-69. doi: 10.1302/2046-3758.65.bjr-2017-0330.r1 | No | No | Yes |
| McLeod A, Weir A, Aitken C, et al. Rise in testing and diagnosis associated with Scotland's Action Plan on Hepatitis C and introduction of dried blood spot testing. Journal of Epidemiology and Community Health 2014;68(12):1182-88. doi: 10.1136/jech-2014-204451 | No | Yes | Yes |
| McLintock K, Russell AM, Alderson SL, et al. The effects of financial incentives for case finding for depression in patients with diabetes and coronary heart disease: interrupted time series analysis. BMJ Open 2014;4(8):e005178-e78. doi: 10.1136/bmjopen-2014-005178 | No | No | No |
| McPhedran S, Mauser G. Lethal Firearm-Related Violence Against Canadian Women: Did Tightening Gun Laws Have an Impact on Women’s Health and Safety? Violence and Victims 2013;28(5):875-83. doi: 10.1891/0886-6708.vv-d-12-00145 | No | No | Yes |
| Mead EL, Cruz-Cano R, Bernat D, et al. Association between Florida's smoke-free policy and acute myocardial infarction by race: A time series analysis, 2000–2013. Preventive Medicine 2016;92:169-75. doi: 10.1016/j.ypmed.2016.05.032 | No | No | Yes |
| Meirambayeva A, Vingilis E, Zou G, et al. Evaluation of Deterrent Impact of Ontario's Street Racing and Stunt Driving Law on Extreme Speeding Convictions. Traffic Injury Prevention 2014;15(8):786-93. doi: 10.1080/15389588.2014.890721 | No | No | No |
| Mellon L, Hickey A, Doyle F, et al. Can a media campaign change health service use in a population with stroke symptoms? Examination of the first Irish stroke awareness campaign. Emergency Medicine Journal 2013;31(7):536-40. doi: 10.1136/emermed-2012-202280 | No | No | Yes |
| Melvin KE, Hart JC, Sorvig RD. Second-Generation Antipsychotic Prescribing Patterns for Pediatric Patients Enrolled in West Virginia Medicaid. Psychiatric Services 2017;68(10):1061-67. doi: 10.1176/appi.ps.201600489 | No | No | Yes |
| Milder EA, Rizzi MD, Morales KH, et al. Impact of a New Practice Guideline on Antibiotic Use With Pediatric Tonsillectomy. JAMA Otolaryngology–Head & Neck Surgery 2015;141(5):410. doi: 10.1001/jamaoto.2015.95 | No | No | Yes |
| Miller P, Curtis A, Palmer D, et al. Changes in injury-related hospital emergency department presentations associated with the imposition of regulatory versus voluntary licensing conditions on licensed venues in two cities. Drug and Alcohol Review 2014;33(3):314-22. doi: 10.1111/dar.12118 | No | No | Yes |
| Miwa S, Visintainer P, Engelman R, et al. Effects of an Ambulation Orderly Program Among Cardiac Surgery Patients. The American Journal of Medicine 2017;130(11):1306-12. doi: 10.1016/j.amjmed.2017.04.044 | No | Yes | Yes |
| Moyo P, Simoni-Wastila L, Griffin BA, et al. Impact of prescription drug monitoring programs (PDMPs) on opioid utilization among Medicare beneficiaries in 10 US States. Addiction 2017;112(10):1784-96. doi: 10.1111/add.13860 | No | Yes | Yes |
| Muoto I, Darney BG, Lau B, et al. Shifting Patterns in Cesarean Delivery Scheduling and Timing in Oregon before and after a Statewide Hard Stop Policy. Health Services Research 2017;53:2839-57. doi: 10.1111/1475-6773.12797 | No | No | Yes |
| Myung W, Lee G-H, Won H-H, et al. Paraquat Prohibition and Change in the Suicide Rate and Methods in South Korea. PLOS ONE 2015;10(6):e0128980. doi: 10.1371/journal.pone.0128980 | Yes | N/A | Yes |
| Nakahara S, Ichikawa M, Nakajima Y. Effects of Increasing Child Restraint Use in Reducing Occupant Injuries Among Children Aged 0–5 Years in Japan. Traffic Injury Prevention 2014;16(1):55-61. doi: 10.1080/15389588.2014.897698 | No | No | Yes |
| Narayan H, Thomas SHL, Eddleston M, et al. Disproportionate effect on child admissions of the change in Medicines and Healthcare Products Regulatory Agency guidance for management of paracetamol poisoning: an analysis of hospital admissions for paracetamol overdose in England and Scotland. British Journal of Clinical Pharmacology 2015;80(6):1458-63. doi: 10.1111/bcp.12779 | Yes | N/A | Yes |
| Nazif-Munoz JI, Quesnel-Vallée A, van den Berg A. Did Chile’s traffic law reform push police enforcement? Understanding Chile’s traffic fatalities and injuries reduction. Injury Prevention 2014;21(3):159-65. doi: 10.1136/injuryprev-2014-041358 | No | Yes | Yes |
| Newitt S, Myles PR, Birkin JA, et al. Impact of infection control interventions on rates of Staphylococcus aureus bacteraemia in National Health Service acute hospitals, East Midlands, UK, using interrupted time-series analysis. Journal of Hospital Infection 2015;90(1):28-37. doi: 10.1016/j.jhin.2014.12.016 | No | No | Yes |
| Nistal-Nuño B. Segmented regression analysis of interrupted time series data to assess outcomes of a South American road traffic alcohol policy change. Public Health 2017;150:51-59. doi: 10.1016/j.puhe.2017.04.025 | No | No | Yes |
| Norstrom T, Stickley A. Alcohol tax, consumption and mortality in tsarist Russia: is a public health perspective applicable? The European Journal of Public Health 2012;23(2):340-44. doi: 10.1093/eurpub/cks079 | No | No | Yes |
| O’Brien NP, Foss RD, Goodwin AH, et al. Supervised hours requirements in graduated driver licensing: Effectiveness and parental awareness. Accident Analysis & Prevention 2013;50:330-35. doi: 10.1016/j.aap.2012.05.007 | No | No | Yes |
| Okasha O, Rinta-Kokko H, Palmu AA, et al. Population-level impact of infant 10-valent pneumococcal conjugate vaccination on adult pneumonia hospitalisations in Finland. Thorax 2017;73(3):262-69. doi: 10.1136/thoraxjnl-2017-210440 | No | No | No |
| Osman M, Parnell AC. Effect of the First World War on suicide rates in Ireland: an investigation of the 1864–1921 suicide trends. BJPsych Open 2015;1(2):164-65. doi: 10.1192/bjpo.bp.115.000539 | No | Yes | Yes |
| Ostrowsky B, Sharma S, DeFino M, et al. Antimicrobial Stewardship and Automated Pharmacy Technology Improve Antibiotic Appropriateness for Community-Acquired Pneumonia. Infection Control & Hospital Epidemiology 2013;34(6):566-72. doi: 10.1086/670623 | No | No | Yes |
| Owens CL, Peterson D, Kamineni A, et al. Effects of transitioning from conventional methods to liquid-based methods on unsatisfactory Papanicolaou tests. Cancer Cytopathology 2013;121(10):568-75. doi: 10.1002/cncy.21309 | No | No | Yes |
| Pace LE, Dusetzina SB, Keating NL. Early Impact of the Affordable Care Act on Uptake of Long-acting Reversible Contraceptive Methods. Medical Care 2016;54(9):811-17. doi: 10.1097/mlr.0000000000000551 | No | No | Yes |
| Pan SW, Chong HH, Kao H-C. Unintentional injury mortality among indigenous communities of Taiwan: trends from 2002 to 2013 and evaluation of a community-based intervention. Injury Prevention 2017;25(1):26-30. doi: 10.1136/injuryprev-2017-042321 | No | Yes | Yes |
| Panagiotoglou D, Law MR, McGrail K. Effect of Hospital Closures on Acute Care Outcomes in British Columbia, Canada. Medical Care 2017;55(1):50-56. doi: 10.1097/mlr.0000000000000619 | No | No | Yes |
| Panatto D, Domnich A, Gasparini R, et al. An eHealth Project on Invasive Pneumococcal Disease: Comprehensive Evaluation of a Promotional Campaign. Journal of Medical Internet Research 2016;18(12):e316. doi: 10.2196/jmir.6205 | No | Yes | No |
| Parikh K, Hall M, Teach SJ. Bronchiolitis Management Before and After the AAP Guidelines. Pediatrics 2013;133(1):e1-e7. doi: 10.1542/peds.2013-2005 | No | No | No |
| Patel PR, Yi SH, Booth S, et al. Bloodstream Infection Rates in Outpatient Hemodialysis Facilities Participating in a Collaborative Prevention Effort: A Quality Improvement Report. American Journal of Kidney Diseases 2013;62(2):322-30. doi: 10.1053/j.ajkd.2013.03.011 | No | No | Yes |
| Pegues DA, Han J, Gilmar C, et al. Impact of Ultraviolet Germicidal Irradiation for No-Touch Terminal Room Disinfection on Clostridium difficile Infection Incidence Among Hematology-Oncology Patients. Infection Control & Hospital Epidemiology 2016;38(1):39-44. doi: 10.1017/ice.2016.222 | No | Yes | Yes |
| Pellegrin KL, Krenk L, Oakes SJ, et al. Reductions in Medication-Related Hospitalizations in Older Adults with Medication Management by Hospital and Community Pharmacists: A Quasi-Experimental Study. Journal of the American Geriatrics Society 2016;65(1):212-19. doi: 10.1111/jgs.14518 | No | No | Yes |
| Petereit D, Omidpanah A, Boylan A, et al. A Multi-faceted Approach to Improving Breast Cancer Outcomes in a Rural Population, and the Potential Impact of Patient Navigation. South Dakota medicine : the journal of the South Dakota State Medical Association 2016;69(6):268-73. [published Online First: 2016/07/23] | No | No | Yes |
| Petrou P. The Ariadne's thread in co-payment, primary health care usage and financial crisis: findings from Cyprus public health care sector. Public Health 2015;129(11):1503-09. doi: 10.1016/j.puhe.2015.07.032 | No | No | Yes |
| Pinheiro SP, Kang EM, Kim CY, et al. Concomitant use of isotretinoin and contraceptives before and after iPledge in the United States. Pharmacoepidemiology and Drug Safety 2013;22(12):1251-57. doi: 10.1002/pds.3481 | No | No | Yes |
| Poluzzi E, Veronese G, Piccinni C, et al. Switching among Equivalents in Chronic Cardiovascular Therapies: ‘Real World’ Data from Italy. Basic & Clinical Pharmacology & Toxicology 2015;118(1):63-69. doi: 10.1111/bcpt.12442 | No | Yes | Yes |
| Pradhan A, Anasuya A, Pradhan MM, et al. Trends in Malaria in Odisha, India—An Analysis of the 2003–2013 Time-Series Data from the National Vector Borne Disease Control Program. PLOS ONE 2016;11(2):e0149126. doi: 10.1371/journal.pone.0149126 | No | No | No |
| Pridemore WA, Chamlin MB, Andreev E. Reduction in Male Suicide Mortality Following the 2006 Russian Alcohol Policy: An Interrupted Time Series Analysis. American Journal of Public Health 2013;103(11):2021-26. doi: 10.2105/ajph.2013.301405 | No | No | Yes |
| Prinja S, Kaur G, Gupta R, et al. Out-of-pocket expenditure for health care: District level estimates for Haryana state in India. The International Journal of Health Planning and Management 2018;34(1):277-93. doi: 10.1002/hpm.2628 | No | No | Yes |
| Puig-Junoy J, Rodríguez-Feijoó S, Lopez-Valcarcel BG. Paying for Formerly Free Medicines in Spain After 1 Year of Co-Payment: Changes in the Number of Dispensed Prescriptions. Applied Health Economics and Health Policy 2014;12(3):279-87. doi: 10.1007/s40258-014-0097-6 | No | Yes | No |
| Pun VC, Lin H, Kim JH, et al. Impacts of alcohol duty reductions on cardiovascular mortality among elderly Chinese: a 10-year time series analysis. Journal of Epidemiology and Community Health 2013;67(6):514-18. doi: 10.1136/jech-2012-201859 | No | No | Yes |
| Rhodes D, Cheng AC, McLellan S, et al. Reducing Staphylococcus aureus bloodstream infections associated with peripheral intravenous cannulae: successful implementation of a care bundle at a large Australian health service. Journal of Hospital Infection 2016;94(1):86-91. doi: 10.1016/j.jhin.2016.05.020 | No | No | No |
| Rooholamini SN, Clifton H, Haaland W, et al. Outcomes of a Clinical Pathway to Standardize Use of Maintenance Intravenous Fluids. Hospital Pediatrics 2017;7(12):703-09. doi: 10.1542/hpeds.2017-0099 | No | Yes | Yes |
| Rosenthal MB, Friedberg MW, Singer SJ, et al. Effect of a Multipayer Patient-Centered Medical Home on Health Care Utilization and Quality. JAMA Internal Medicine 2013;173(20):1907. doi: 10.1001/jamainternmed.2013.10063 | No | Yes | Yes |
| Rutman L, Wright DR, OʼCallaghan J, et al. A Comprehensive Approach to Pediatric Pneumonia. Journal for Healthcare Quality 2017;39(4):e59-e69. doi: 10.1097/jhq.0000000000000048 | No | No | Yes |
| Ryu S, Lau CL, Chun BC. The impact of Livestock Manure Control Policy on human leptospirosis in Republic of Korea using interrupted time series analysis. Epidemiology and Infection 2017;145(7):1320-25. doi: 10.1017/s0950268817000218 | No | No | Yes |
| Sakai R, Wang W, Yamaguchi N, et al. The Impact of Japan's 2004 Postgraduate Training Program on Intra-Prefectural Distribution of Pediatricians in Japan. PLoS ONE 2013;8(10):e77045. doi: 10.1371/journal.pone.0077045 | No | No | Yes |
| Santa-Ana-Tellez Y, Mantel-Teeuwisse AK, Dreser A, et al. Impact of Over-the-Counter Restrictions on Antibiotic Consumption in Brazil and Mexico. PLoS ONE 2013;8(10):e75550. doi: 10.1371/journal.pone.0075550 | No | No | Yes |
| Santa-Ana-Tellez Y, Mantel-Teeuwisse AK, Leufkens HGM, et al. Seasonal Variation in Penicillin Use in Mexico and Brazil: Analysis of the Impact of Over-the-Counter Restrictions. Antimicrobial Agents and Chemotherapy 2014;59(1):105-10. doi: 10.1128/aac.03629-14 | No | No | Yes |
| Scherb HH, Mori K, Hayashi K. Increases in perinatal mortality in prefectures contaminated by the Fukushima nuclear power plant accident in Japan. Medicine 2016;95(38):e4958. doi: 10.1097/md.0000000000004958 | No | No | Yes |
| Sicsic J, Saint-Lary O, Rouveix E, et al. Impact of a primary care national policy on HIV screening in France: a longitudinal analysis between 2006 and 2013. British Journal of General Practice 2016;66(653):e920-e29. doi: 10.3399/bjgp16x687529 | No | Yes | Yes |
| Singh K, Speizer I, Handa S, et al. Impact evaluation of a quality improvement intervention on maternal and child health outcomes in Northern Ghana: early assessment of a national scale-up project. International Journal for Quality in Health Care 2013;25(5):477-87. doi: 10.1093/intqhc/mzt054 | No | No | Yes |
| Sinnott S-J, Normand C, Byrne S, et al. Copayments for prescription medicines on a public health insurance scheme in Ireland. Pharmacoepidemiology and Drug Safety 2015;25(6):695-704. doi: 10.1002/pds.3917 | No | No | No |
| Slattery C, Freund M, Gillham K, et al. Increasing smoking cessation care across a network of hospitals: an implementation study. Implementation Science 2015;11(1) doi: 10.1186/s13012-016-0390-x | No | No | Yes |
| Smith RL, Hayashi VN, Lee YI, et al. The Medical Emergency Team Call. Critical Care Medicine 2014;42(2):322-27. doi: 10.1097/ccm.0b013e3182a27413 | No | No | Yes |
| Snowden LR, Wallace N, Cordell K, Graaf G. Increased mental health treatment financing, community-based organization’s treatment programs, and Latino-White children’s financing disparities. Journal of Mental Health Policy and Economics 2017;20:137-145. | No | No | No |
| Staras SAS, Livingston MD, Christou AM, et al. Heterogeneous population effects of an alcohol excise tax increase on sexually transmitted infections morbidity. Addiction 2014;109(6):904-12. doi: 10.1111/add.12493 | No | Yes | Yes |
| Stelfox HT, Bastos J, Niven DJ, et al. Critical care transition programs and the risk of readmission or death after discharge from ICU. Intensive Care Medicine 2016;42(3):401-10. doi: 10.1007/s00134-015-4173-7 | No | No | Yes |
| Taber DJ, DuBay D, McGillicuddy JW, et al. Impact of the New Kidney Allocation System on Perioperative Outcomes and Costs in Kidney Transplantation. Journal of the American College of Surgeons 2017;224(4):585-92. doi: 10.1016/j.jamcollsurg.2016.12.009 | No | Yes | Yes |
| Thijssen WAMH, Wijnen-van Houts M, Koetsenruijter J, et al. The Impact on Emergency Department Utilization and Patient Flows after Integrating with a General Practitioner Cooperative: An Observational Study. Emergency Medicine International 2013;2013:1-8. doi: 10.1155/2013/364659 | No | Yes | Yes |
| Tiwari A, Osbert N, Matimelo SM, et al. Assessing the Impact of Leveraging Traditional Leadership on Access to Sanitation in Rural Zambia. The American Journal of Tropical Medicine and Hygiene 2017;97(5):1355-61. doi: 10.4269/ajtmh.16-0612 | No | Yes | No |
| Troelstra S, Bosdriesz J, de Boer M, et al. Effect of tobacco control policies on information seeking for smoking cessation in the Netherlands: A Google Trends study. European Journal of Public Health 2014;24(suppl_2) doi: 10.1093/eurpub/cku164.043 | No | Yes | No |
| Ullman M, Parlier G, Warren J, et al. The Economic Impact of Starting, Stopping, and Restarting an Antibiotic Stewardship Program: A 14-Year Experience. Antibiotics 2013;2(2):256-64. doi: 10.3390/antibiotics2020256 | No | No | No |
| Yarnell CJ, Shadowitz S, Redelmeier DA. Hospital Readmissions Following Physician Call System Change: A Comparison of Concentrated and Distributed Schedules. The American Journal of Medicine 2016;129(7):706-14.e2. doi: 10.1016/j.amjmed.2016.02.022 | No | Yes | Yes |
| Zhao A, Chen R, Qi Y, et al. Evaluating the Impact of Criminalizing Drunk Driving on Road-Traffic Injuries in Guangzhou, China: A Time-Series Study. Journal of Epidemiology 2016;26(8):433-39. doi: 10.2188/jea.je20140103 | No | No | No |
