# Supplementary material for: Comparison of six statistical methods for interrupted time series studies: empirical evaluation of 190 published series
Source: BMC Med Res Methodol. 2021 Jun 26;21:134. doi: 10.1186/s12874-021-01306-w (PMC8235830; doi:10.1186/s12874-021-01306-w)
Supplement: Supplementary file 3 — Additional file 3: Appendix 1: Interrupted time series with a transition period. Appendix 2: Difference in level and slope change by length of time series. Appendix 3: Standardising the direction of effect. Appendix 4: Detailed p-value comparisons. [file 12874_2021_1306_MOESM3_ESM.docx]

# Appendices

The following sections contain Appendices to the study: “Comparison of six statistical methods for interrupted time series studies: empirical evaluation of 190 published series”

Simon L Turner^1^, Amalia Karahalios^1^, Andrew B Forbes^1^, Monica Taljaard^2,3^, Jeremy M Grimshaw^2,3,4^, Joanne E McKenzie^1*^

^1^School of Public Health and Preventive Medicine, Monash University, Melbourne, Victoria, Australia.

^2^Clinical Epidemiology Program, Ottawa Hospital Research Institute, Ottawa, Ontario, Canada. 1053 Carling Ave, Ottawa.

^3^School of Epidemiology and Public Health, University of Ottawa, Ottawa, Ontario, Canada. 600 Peter Morand Crescent, Ottawa, Ontario K1G 5Z3.

^4^Department of Medicine, University of Ottawa, Ottawa, Ontario, Canada. Roger Guindon Hall, 451 Smyth Rd.

## Appendix 1: Interrupted time series with a transition period

When a transition period was included the level change was measured from the counterfactual to the post-transition period Figure A1).


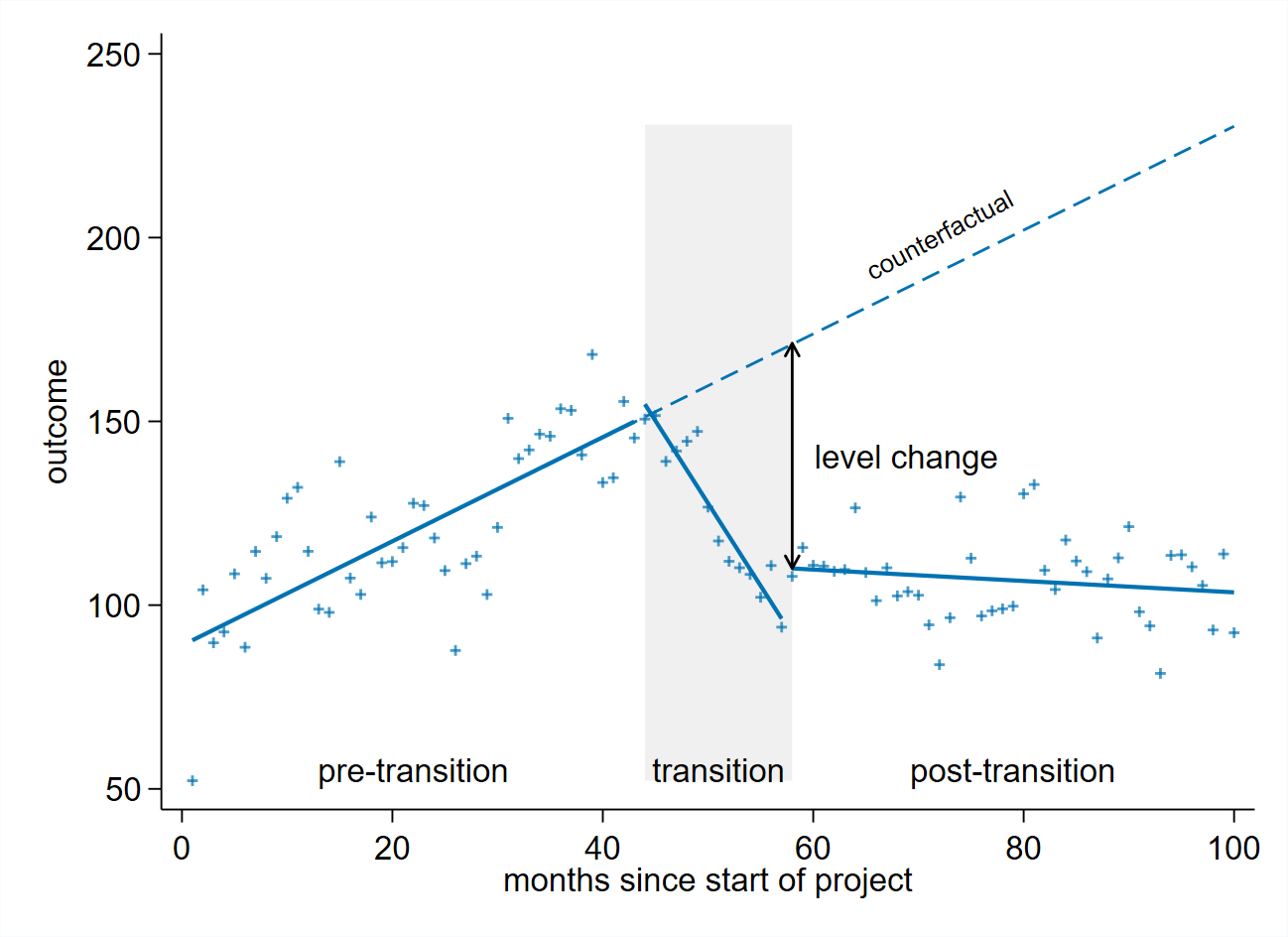

Figure A1: Interrupted time series with a transition period. The level change is calculated as the vertical distance between the counterfactual trend line and the post-transition trend line at the beginning of the post-transition series. The slope change is calculated as the change in slope between the counterfactual trend line and post-transition trend line.

## Appendix 2: Difference in level and slope change by length of time series

We investigated the impact of series length on the difference in level and slope change estimates between each pair of methods. A matrix of scatterplots of the differences in level change (or slope change) versus the (log) length of series (overlaid with a local regression (LOESS) smoothed curve) for each pairwise method comparison is presented in Figure A2.


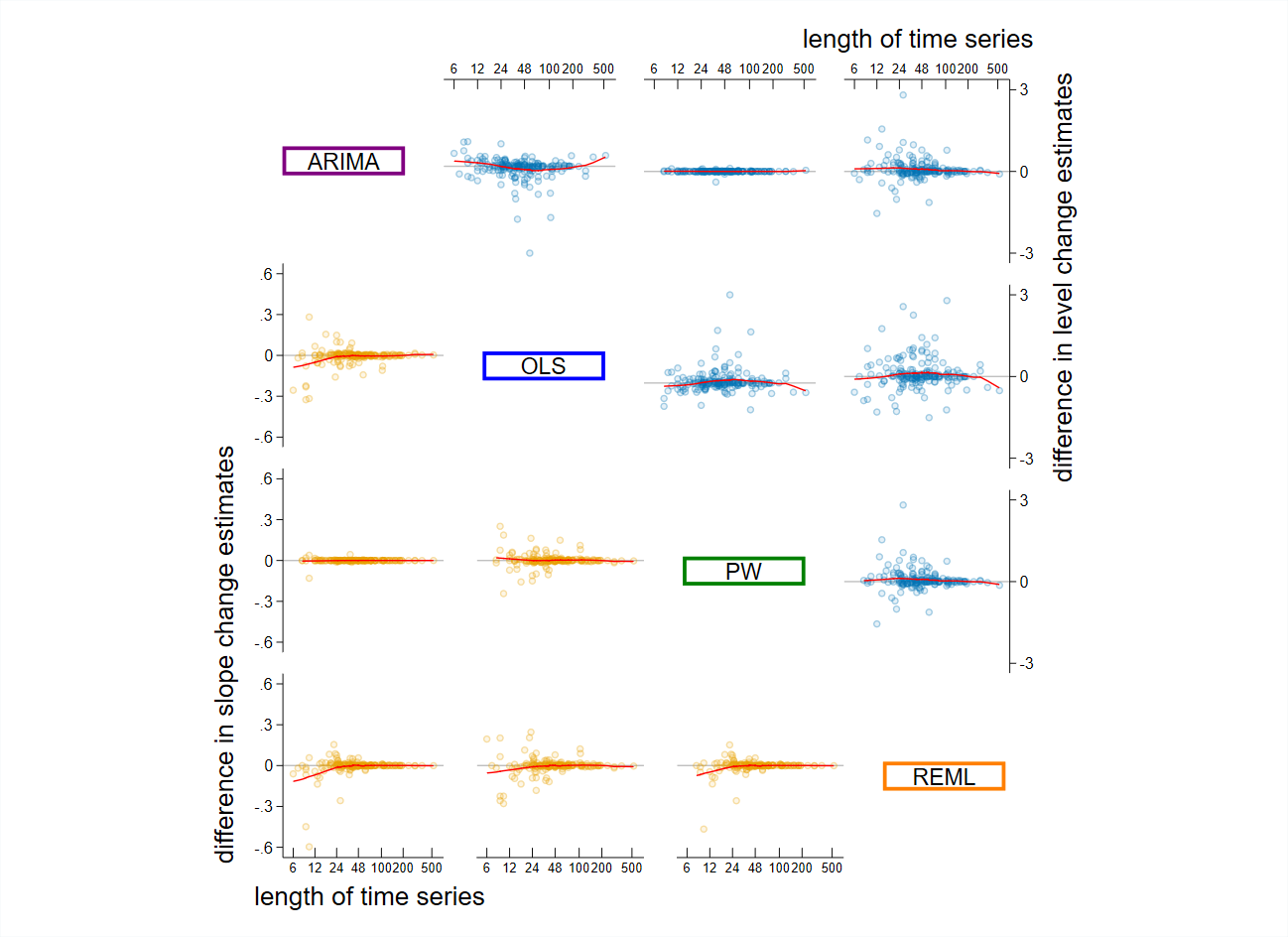


Figure A2: Scatter plot of standardised effect estimate differences versus series length. Blue circles (top triangle) show difference in level change estimates, orange circles (bottom triangle) show difference in slope change estimates.The vertical axis shows the effect estimate of the row method – the effect estimate of the column method. The horizontal axis shows the length of the time series (using a log scale). Red lines depict local regression (LOESS) smoothed curves. Grey lines indicate zero. Abbreviations: ARIMA, autoregressive integrated moving average; OLS, ordinary least squares; PW, Prais-Winsten; REML, restricted maximum likelihood. Note that NW and REML-Satt are not shown as the NW and OLS methods use the same estimator for level and slope change, as do REML and REML-Satt, which also use the same estimator for standard errors.

## Appendix 3: Standardising the direction of effect

We standardised the direction of effect for each pairwise comparison of methods by multiplying both estimates by -1 if the first method’s estimate was less than zero. Given the choice of first method was arbitrary, we repeated these analyses by standardising to the direction of the second method’s estimate. Table A1 presents equivalent information to that presented in the top triangles of Tables 5 and 6, except with the standardisation reversed. The direction of standardisation did not affect mean differences in slope change estimates between methods, and had a small, but unimportant, impact for level change.

Table A1: Mean of differences in level change estimates between methods (column method - row method) (top triangle) and geometric mean ratio of standard errors for level change between methods (row method/column method) (shaded bottom triangle) with 95% limits of agreement. The NW and OLS methods use the same estimator for level and slope change, as do REML and REML-Satt (not shown), which also use the same estimator for standard errors.

| **Level Change** | **Mean of differences in level change estimates between methods (95% limits of agreement)** | | | | |
| --- | --- | --- | --- | --- | --- |
| **Mean of differences in slope change estimates between methods (95% limits of agreement)** | **ARIMA** | 0.05 (-0.81,0.92) | 0.05 (-0.81,0.92) | 0.00 (-0.07,0.07) | -0.11 (-0.86,0.64) |
|  | -0.01 (-0.12,0.11) | **OLS** | - | -0.09 (-0.97,0.78) | -0.17 (-1.25,0.91) |
|  | -0.01 (-0.12,0.11) | - | **NW** | -0.09 (-0.97,0.78) | -0.17 (-1.25,0.91) |
|  | 0.00 (-0.02,0.02) | 0.00 (-0.09,0.09) | 0.00 (-0.09,0.09) | **PW** | -0.11 (-0.84,0.63) |
|  | -0.01 (-0.13,0.12) | 0.00 (-0.12,0.12) | 0.00 (-0.12,0.12) | 0.00 (-0.10,0.09) | **REML** |

## Appendix 4: Detailed p-value comparisons

Pairwise comparisons of p-values for each series are shown in detail for both level and slope change in Figure A3: Pairwise comparisons of p-values between all statistical methods. The top triangle refers to level change p-values, the bottom triangle refers to slope change p-values. Dashed red lines indicate p-values of 0.05, dashed grey lines indicate p-values of 0.01. Abbreviations: ARIMA, autoregressive integrated moving average; OLS, ordinary least squares; NW OLS with Newey-West standard error adjustments; PW, Prais-Winsten; REML, restricted maximum likelihood; Satt, Satterthwaite adjustment..


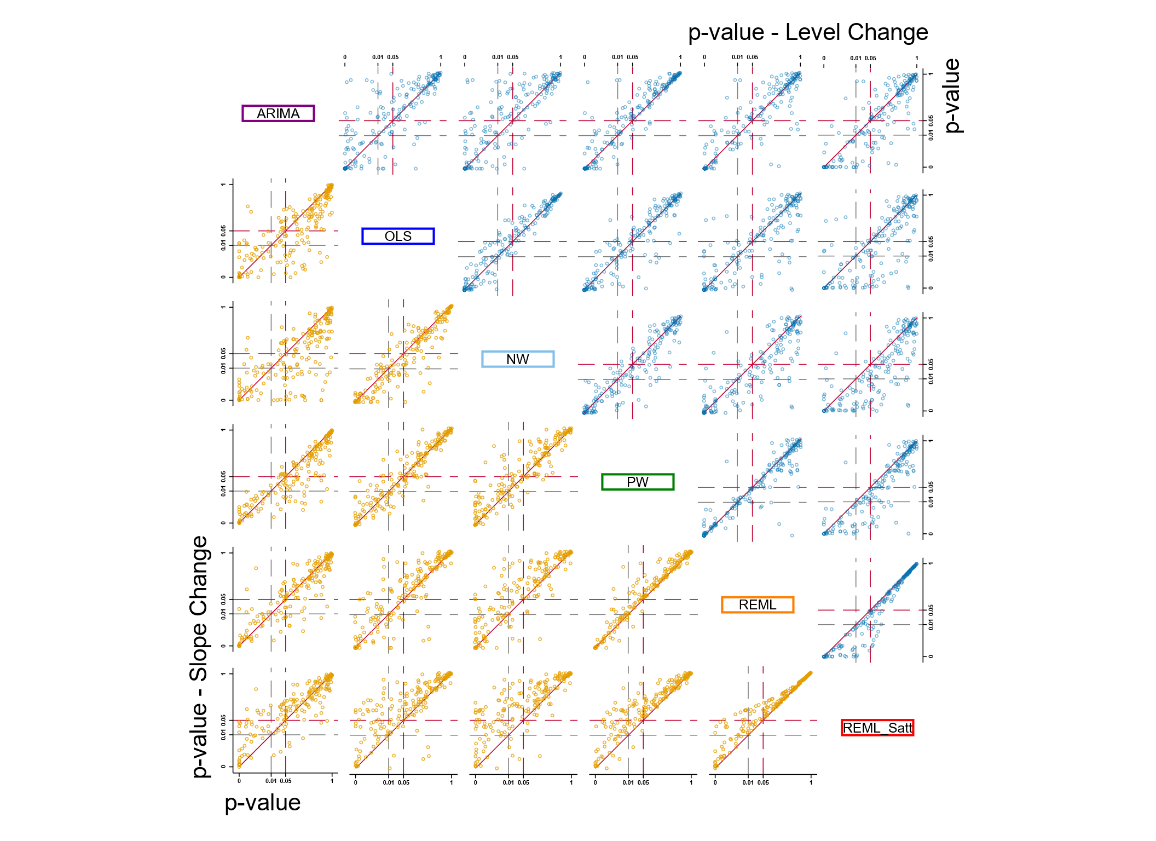

Figure A3: Pairwise comparisons of p-values between all statistical methods. The top triangle refers to level change p-values, the bottom triangle refers to slope change p-values. Dashed red lines indicate p-values of 0.05, dashed grey lines indicate p-values of 0.01. Abbreviations: ARIMA, autoregressive integrated moving average; OLS, ordinary least squares; NW OLS with Newey-West standard error adjustments; PW, Prais-Winsten; REML, restricted maximum likelihood; Satt, Satterthwaite adjustment.
